# Targeting cancer stem cell OXPHOS with tailored ruthenium complexes as a new anti-cancer strategy

**DOI:** 10.1186/s13046-023-02931-7

**Published:** 2024-01-27

**Authors:** Sonia Alcalá, Lara Villarino, Laura Ruiz-Cañas, José R. Couceiro, Miguel Martínez-Calvo, Adrián Palencia-Campos, Diego Navarro, Pablo Cabezas-Sainz, Iker Rodriguez-Arabaolaza, Alfonso Cordero-Barreal, Lucia Trilla-Fuertes, Juan A. Rubiolo, Sandra Batres-Ramos, Mireia Vallespinos, Cristina González-Páramos, Jéssica Rodríguez, Angelo Gámez-Pozo, Juan Ángel Fresno Vara, Sara Fra Fernández, Amparo Benito Berlinches, Nicolás Moreno-Mata, Ana María Torres Redondo, Alfredo Carrato, Patrick C. Hermann, Laura Sánchez, Susana Torrente, Miguel Ángel Fernández-Moreno, José L. Mascareñas, Bruno Sainz

**Affiliations:** 1 Department of Biochemistry, Autónoma University of Madrid, School of Medicine and Department of Cancer, Instituto de Investigaciones Biomédicas (IIBm) Sols-Morreale (CSIC-UAM), Madrid, Spain; 2 Biomarkers and Personalized Approach to Cancer (BIOPAC) Group, Area 3 Cancer, Instituto Ramón y Cajal de Investigación Sanitaria (IRYCIS), Madrid, Spain; 3 Centro Singular de Investigación en Química Biolóxica e Materiais Moleculares (CIQUS), and Departamento de Química Orgánica, Universidade de Santiago de Compostela (USC), Santiago de Compostela, Spain; 4 Facultad de Ciencia y Técnología, Universidad del País Vasco, 48940 Leioa (Bizkaia), Spain; 5 Department of Zoology, Genetics and Physical Anthropology, Veterinary Faculty, USC, Lugo, Spain; 6 Molecular Oncology and Pathology Lab, Instituto de Genética Médica y Molecular-INGEMM, Instituto de Investigación Hospital Universitario La Paz-IdiPAZ, Madrid, Spain; 7 Biomedica Molecular Medicine SL, Madrid, Spain; 8 Centro de Investigación Biomédica en Red en Enfermedades Raras (CIBERER), Madrid, Spain; 9 Instituto de Investigación Sanitaria Hospital 12 de Octubre (imas12), Madrid, Spain; 10 Centro de Investigación Biomédica en Red, Área Cáncer, CIBERONC, ISCIII, Madrid, Spain; 11 Servicio de Cirugía Torácica, Hospital Universitario Ramón y Cajal, Madrid, Spain; 12 Servicio de Anatomía Patológica, Hospital Universitario Ramón y Cajal, Madrid, Spain; 13 Biobanco Hospital Universitario Ramón y Cajal, IRYCIS, Madrid, Spain; 14 Pancreatic Cancer Europe (PCE) Chairperson, Brussels, Belgium; 15 Department of Internal Medicine I, Ulm University, Ulm, Germany; 16 Valuation, Transfer and Entrepreneurship Area, USC, Santiago de Compostela, Spain; 17 Department of Biochemistry, Autónoma University of Madrid, School of Medicine and Department of Rare Diseases, Instituto de Investigaciones Biomédicas (IIBm) Sols-Morreale (CSIC-UAM), Madrid, Spain

**Keywords:** Pancreatic ductal adenocarcinoma, Cancer stem cells, Ruthenium complexes, Mitochondrial DNA, Anti-cancer agents, Oxidative phosphorylation, Patient-derived xenografts, Colon cancer

## Abstract

**Background:**

Previous studies by our group have shown that oxidative phosphorylation (OXPHOS) is the main pathway by which pancreatic cancer stem cells (CSCs) meet their energetic requirements; therefore, OXPHOS represents an Achille’s heel of these highly tumorigenic cells. Unfortunately, therapies that target OXPHOS in CSCs are lacking.

**Methods:**

The safety and anti-CSC activity of a ruthenium complex featuring bipyridine and terpyridine ligands and one coordination labile position (*Ru1*) were evaluated across primary pancreatic cancer cultures and in vivo, using 8 patient-derived xenografts (PDXs). RNAseq analysis followed by mitochondria-specific molecular assays were used to determine the mechanism of action.

**Results:**

We show that *Ru1* is capable of inhibiting CSC OXPHOS function in vitro, and more importantly, it presents excellent anti-cancer activity, with low toxicity, across a large panel of human pancreatic PDXs, as well as in colorectal cancer and osteosarcoma PDXs. Mechanistic studies suggest that this activity stems from *Ru1* binding to the D-loop region of the mitochondrial DNA of CSCs, inhibiting OXPHOS complex-associated transcription, leading to reduced mitochondrial oxygen consumption, membrane potential, and ATP production, all of which are necessary for CSCs, which heavily depend on mitochondrial respiration.

**Conclusions:**

Overall, the coordination complex *Ru1* represents not only an exciting new anti-cancer agent, but also a molecular tool to dissect the role of OXPHOS in CSCs. Results indicating that the compound is safe, non-toxic and highly effective in vivo are extremely exciting, and have allowed us to uncover unprecedented mechanistic possibilities to fight different cancer types based on targeting CSC OXPHOS.

**Supplementary Information:**

The online version contains supplementary material available at 10.1186/s13046-023-02931-7.

## Background

Despite significant advances in both our understanding of cancer, and in the development of new treatments, annual world-wide cancer-related fatalities remain high, with 9.6 million deaths (accounting for 1 in 6 deaths) alone in 2018 (World Health Organization). This is particularly true for tumors driven by cancer stem cells (CSCs), which have been shown to be responsible for tumor heterogeneity, metastasis, chemoresistance, and tumor relapse in a large number of cancers [1–4]. Thus, from a medicinal perspective, targeting the CSC population represents a very appealing anti-cancer strategy. Unfortunately, progress in the development of anti-CSC agents has been very slow, and these types of compounds are still very far from reaching the clinic (reviewed in [5–7]).

We and others have studied CSCs at the genetic, epigenetic, transcriptional, proteome and metabolic levels to identify their targetable weaknesses [8–11], allowing us to discover that CSCs of many tumor types [12, 13], such as pancreatic ductal adenocarcinoma (PDAC), preferentially use aerobic oxidative phosphorylation (OXPHOS) over anaerobic glycolysis to meet their energy requirements. As a consequence, CSCs exhibit increased mitochondrial mass and membrane potential (reflection of mitochondrial function) [14]. While OXPHOS involves a significantly greater number of biochemical reactions, it is almost 20 times more efficient than glycolysis in terms of generation of ATP per unit of glucose. Considering this dependence on mitochondrial respiration, it is reasonable to predict that targeting OXPHOS in CSCs may represent an effective approach for treating cancer.

Some of us have recently demonstrated that ruthenium complexes containing a bipyridine and a terpyridine ligand, and one exchangeable (reactive) coordinating position ([Ru(terpy)(bpy)X]^n+^) can react with solvent accessible guanines in DNA [15, 16]. In contrast to other standard DNA metalating agents, such as cisplatin, these types of complexes present a kinetically controlled reactivity with DNA, likely due to the bulkiness provided by their ruthenium ligands, which also allows for exquisite chemoselectivity. Likewise, these complexes were found to smoothly ruthenate solvent-exposed guanines present in adjacent positions of four-stranded guanine DNA quadruplexes (GQs) [15]. GQs and related secondary DNA structures play important physiological roles in controlling telomere association, recombination and replication, or in regulating transcription [17]. These structures are present in nuclear DNA, but can also be found throughout the mitochondrial DNA (mtDNA), where they can contribute to the regulation of mitochondrial gene expression [18], and thus cellular metabolism and respiratory functions [19].

Considering the smooth reactivity of the aforementioned ruthenium complexes with DNA secondary structures, and given that their lipophilic and positively charged nature may facilitate a mitochondrial accumulation [20–22], we questioned whether they could target the mtDNA of CSCs and thereby affect OXPHOS. This might eventually translate into compelling anticancer effects.

Using pancreatic CSCs (PaCSCs) as a model system, we herein demonstrate that ruthenium complexes of type ([Ru(terpy)(bpy)X]^n+^) present a remarkable ability to reduce the self-renewal, invasive and tumorigenic capacity of PaCSCs by shutting down the transcription of their mtDNA protein-encoding genes and compromising their OXPHOS-dependent respiration. In contrast to other reported bioactive metal complexes [21, 22], our compounds are not cytotoxic, and do not induce ROS or apoptosis. Our current data suggest that the biological effect is associated to a metalating interaction of the ruthenium complexes with specific guanines present in the D-loop region of the mtDNA, the regulatory region for mtDNA replication and transcription. More importantly, the ruthenium complexes exhibited an impressive effect to halt and even reduce tumor growth of pancreatic and colon cancer patient-derived xenografts (PDXs) in pre-clinical *in vivo* models. We also present preliminary data which demonstrate activity for the treatment against an osteosarcoma (OS) PDX.

In brief, we have unveiled a new anti-cancer approach based on targeting key mitochondrial functions of CSCs, and validated the preclinical potential of designed ruthenium complexes in three different types of tumor entities.

## Methods

### Synthesis of ruthenium complexes – General

Chemical synthesis procedures, detailed protocols and characterization of all the compounds are described below (see also Fig. S1 and Fig. 5). NMR and high-resolution electrospray ionization mass spectrometry (HR-ESI-MS) analysis was performed on all the synthesized compounds used in this article.

### Synthesis of the parent ruthenium chloride *[Ru(terpy)(bpy)Cl]Cl (Ru0)*

RuCl3 • 3 H_2_O (2 g, 7.65 mmol) and 2,2’:6’,2’’ terpyridine (1.78 g, 1 equiv) were dissolved in EtOH:H_2_O 1:1 (80 mL), and the mixture was heated under reflux over 4 h in the dark. The resulting precipitate was washed with EtOH (x 3) and Et_2_O (x 1) to give Ru(terpy)Cl_3_ in a 75% yield (2.53 g, 5.74 mmol). The brown solid was used directly in a second step. Ru(terpy)Cl_3_ (550 mg, 1.25 mmol), 2,2’-bipyridine (195.2 mg, 1 equiv) and NEt_3_ (0.52 mL) were dissolved in EtOH:H_2_O 3:1 (120 mL) and the resulting solution was heated under reflux for 4h. The reaction mixture was filtered, and the solvents removed under reduced pressure to near dryness (~ 30 mL). The solution was refrigerated for 48h and the resulting precipitate was collected and washed with Et_2_O (x 1) to give [Ru(terpy)(bpy)Cl]Cl salt (*Ru0*) as a brownish red powder in a 60% yield (421 mg, 0.75 mmol).

### Synthesis of the ruthenium aquo complex Ru1

Aqueous solutions of complex [Ru(terpy)(bpy)Cl]Cl salt (*Ru0*) (either 1 or 5mM) were irradiated with blue LED light (455nm, 40-50W) for 1-2 hours to give the aquo derivative complex [Ru(terpy)(bpy)(H_2_O)]Cl_2_ (*Ru1*). Concentration of the aqueous solutions of complex [Ru(terpy)(bpy)(H_2_O)]Cl_2_ were calculated by using UV-VIS absorption measures [λ_max_ (477 nm) ε = 9600] [23].

### Cell lines, primary human PDAC cells, patient samples

PDAC PDXs were obtained from Dr. Manuel Hidalgo under a Material Transfer Agreement with the Spanish National Cancer Centre (CNIO), Madrid, Spain (Reference no. I409181220BSMH). All PDAC PDX tumors contained G12D mutations in KRAS as determined by PCR sequencing as described in [24]. To establish low-passage primary PDX-derived *in vitro* cultures, PDX tumors were minced, enzymatically digested with collagenase (Stem Cell Technologies) for 60 min at 37°C, clarified via multiple rounds of filter purification with 100µm and 40µm Fisherbrand™ Sterile Cell Strainers (FisherScientific, Cat no. 11517532 and 11587522), and after centrifugation for 5 min at 1800 rpm, the cell pellets were resuspended and cultured in RPMI (Invitrogen) supplemented with 10% FBS (Invitrogen), 50 units/ml penicillin/streptomycin and fungizone (Invitrogen). PDX-derived cultures are referred to by a random number designation (e.g., Panc185, PancA6L, Panc215, Panc253, Panc265 or Panc354). Primary cultures were tested for Mycoplasma at least every 4 weeks.

CRC01 and OS170921 were obtained via the Hospital Ramón y Cajal-IRYCIS BioBank (PT13/0010/0002), integrated in the Spanish National Biobanks Network, under the RG-BIOB-54 Transfer Requests nº208 and nº198 and MTAs AC179 and AC168, and processed following standard operating procedures with the appropriate approval of the Ethical and Scientific Committees (Dictum 140/22 and 280/22), with informed consent and according to Declaration of Helsinki principles. CRC01 and OS170921 were subcutaneously implanted in immunocompromised female 6-week-old NU-Foxn1nu nude mice (Janvier, France) and passaged *in vivo* to establish PDX CRC01 and PDX OS170921.

### Cellular toxicity assay

*Ru1-*mediated cellular toxicity was determined using the Toxilight BioAssay kit (Lonza, Walkersville, MD) according to the manufacturer's instructions.

### Sphere formation assay

Pancreatic CSC spheres were generated by culturing primary pancreatic cancer cells (5,000-20,000 cells/ml) in ultra-low attachment plates (Corning) using serum-free DMEM/F12 (Invitrogen) supplemented with B27 1:50 (Invitrogen), 20ng/mL bFGF (PAN-Biotech) and 50 U/mL penicillin/streptomycin (Thermo Fisher Scientific). Seven days later, spheres were harvested for subsequent assays or counted with an inverted EVOS FL microscope (Thermo Fisher Scientific) using a 10X objective with phase contrast. For serial passaging, spheres were harvested using a 40µm cell strainer (Fisher), trypsinized into single cells and re-cultured for another 7 days. Sphere counts are represented as number (no.) of spheres/ml or the fold change in spheres no./ml.

### Colony assay

For colony formation assays, 500 cells were seeded in 24-well plates. *Ru1* was added 24-48 h post seeding. Cells were cultured in RPMI 1640 containing 10% FBS at 37°C, 5% CO2. After 10-12 days, cells were fixed with PFA 4% (Paraformaldehyde, 16% w/v aq. soln., methanol free, Alfa Aesar™, Cat no. 11400580) for 10 min, washed with PBS and stained with Crystal violet (Sigma, Cat no. C3886-100G) for 1 h. Wells were digitalized and colonies/total area were quantified by lysing stained colonies in 1XPBS with 1%SDS followed by colorimetric absorbance analysis using a Synergy™ HT Multi-Mode Microplate Reader (BioTek, Winooski, Vermont, USA).

### Flow cytometry and FACS

Cells or digested tumors (as described above) were resuspended in Flow buffer [1X PBS; 3% FBS (v/v); 3mM EDTA (v/v)] before analysis with a 4-laser Attune NxT Acoustic Cytometer (Thermo Fisher Scientific). For cell surface marker expression, refer to antibodies listed in Supplementary Table S1. For Annexin-V staining, floating and attached cells were pooled and resuspended in 1X Annexin-V staining buffer containing Annexin-V-FITC diluted 1:20 (Biotium, Freemont, CA) and incubated for 20 min at room temperature prior to flow cytometric analysis. For autofluorescent detection, cells were excited with blue laser 488nm and selected as intersection with emission filters 530/40 (BL1) and 580/30 (BL2) or, in case of sorting, emission filter for FITC. For cell sorting, a FACS Vantage SE Flow Cytometer was used and data analyzed with BD FACSDiVa software.

For mitochondrial membrane potential measurement, CMX-ROS (M7512, Invitrogen), CM-H_2_XRos (M7513, Invitrogen) or Mitoblue [25, 26] were used. Probes were incubated with cells for 20 min at 37°C at a concentration of 10nM, 100nM or 10µM, respectively and fluorescence was detected using the filters (Ex561nm/Em585/16) YL1 for CMX-ROS, (Ex561nm/Em620/15) YL2 for CM-H_2_XRos or (Ex405nm/Em512/25) VL2 for Mitoblue. For mitochondrial mass, 10-N-Nonyl acridine orange (NAO, A7847, Sigma Aldrich) was used at 0.1µM for 20 min at 37°C, and fluorescence was detected using the filters (Ex488nm/Em530/30) BL1. For ROS production measurement, MitoSOX (M36008, Invitrogen) was used at 1µM for 10 min at 37°C and detected with laser (Ex561nm/Em585/16) YL1.

For all assays, 2mg/ml DAPI (Sigma) or 2µl/ml 7-Amino-Actinomycin D (7-AAD, BD, Cat no. 51-68981E) was used to exclude dead cells, and fluorescence was detected using the filters (Ex405nm/Em440/50) VL1 or (Ex561nm/Em695/40) YL3, respectively. Data were analyzed with FlowJo 9.3 software (Tree Star Inc., Ashland, OR.).

### Zebrafish maintenance and xenograft assays

Zebrafish embryos were obtained by mating adult zebrafish (*Danio rerio*, wild-type), maintained in 30L tanks with a ratio of 1 fish per liter of water, with 14h/10h light/dark cycle and a temperature of 28.5°C according to published procedures [27]. All the procedures used in the experiments as well as fish care were performed in agreement with the Animal Care and Use Committee of the University of Santiago de Compostela and the standard protocols of Spain (Directive 2012-63-UE). At the final point of the experiments, zebrafish embryos were euthanized by tricaine overdose.

For zebrafish xenograft assays and image analyses, zebrafish embryos were collected at 0 h post-fertilization (hpf) and incubated until 48 hpf at 28.5°C. At 48 hpf, hatched embryos were anesthetized with 0.003% of tricaine (Sigma). mCherry-H2B-labelled Panc185 cells were treated with *Ru1* (100µM) for 24 h, trypsinized, resuspended and concentrated in an eppendorf at 10^6^ cells per tube for each condition. Cells were then resuspended in 10µL of PBS with 2% PVP (Polyvinylpyrrolidone) to avoid cellular aggregation. Borosilicate needles (1mm O.D. x 0.75mm I.D.; World Precision Instruments) were used to perform the xenograft assays in the zebrafish embryos. Between 100 and 150 cells were injected into the circulation of each fish (Duct of Cuvier) using a microinjector (IM-31 Electric Microinjector, Narishige) with an output pressure of 34 kPA and 30 ms of injection time per injection. Subsequently, the injected embryos were incubated at a temperature of 34°C for 6 dpi in 30ml Petri dishes for each condition with SDTW (salt dechlorinate tap water). Imaging of the injected embryos was performed using a fluorescence stereomicroscope (AZ-100, Nikon) at 1 and/or 6 dpi in order to measure the proliferation, migration and invasion of the Panc185 injected human cancer cells inside the zebrafish circulation in each of the conditions assayed.

The image analysis of the injected embryos was carried out using Quantifish software v2.1 (University College London, London, UK) in order to obtain the proliferation ratio of the cells in the region of the caudal hematopoietic tissue (CHT) of the embryos, where the cells proliferate and metastasize. This program measures in each of the images provided the intensity of the fluorescence and the area of the positive pixel above a certain threshold of the cells. With these parameters, an integrated density value is obtained allowing one to compare different times between images to reach a proliferation ratio.

### In vivo toxicity and tumorigenicity assays

All mice were housed according to institutional guidelines and all experimental procedures were performed in compliance with the institutional guidelines for the welfare of experimental animals approved by the Universidad Autónoma de Madrid Ethics Committee (CEI 60-1057-A068 and CEI 103-1958-A337) and La Comunidad de Madrid (PROEX 335/14 and 294/19) and in accordance with the guidelines for Ethical Conduct in the Care and Use of Animals as stated in The International Guiding Principles for Biomedical Research involving Animals, developed by the Council for International Organizations of Medical Sciences (CIOMS). Briefly, mice were housed according to the following guidelines: a 12 h light/12 h dark cycle, with no access during the dark cycle; temperatures of 65-75°F (~18-23°C) with 40-60% humidity; a standard diet with fat content ranging from 4 to 11%; sterilized water was accessible at all times; for handling, mice were manipulated gently and as little as possible; noises, vibrations and odors were minimized to prevent stress and decreased breeding performance; and enrichment was always used per the facility’s guidelines to help alleviate stress and improve breeding.

For toxicity and preliminary pharmacokinetics (PK) analyses, 10-week-old CD-1 mice (Janvier, France) of approximately 25-30g were treated with *Ru1* via two routes of administration: 1) oral gavage (o.g., 100µl) or 2) retro-orbital (r.o.) injection (100µl). *Ru1* was resuspended in physiological saline (0.45% NaCl) for r.o. injections or in H_2_O for o.g., to a concentration of approximately 0.5mM, such that mice were treated daily with a dose of *Ru1* equivalent to 1.4mg/kg. At indicated time post treatment initiation, mice were weighed. Six and 24h post r.o. injection, and on day 28 (o.g.) or 29 (r.o.), mice were sacrificed, weighed, blood was collected in EDTA tubes (Aquisel, Cat no. 107545) for hematocrit analysis (Element HT5, Veterinary Hematology Analyzer, scil animal care company GmbH, Madrid, Spain), and organs were excised and weighed, photographed, fixed in 4% PFA and processed for histological analysis or analyzed by inductively coupled plasma mass spectrometry (ICP-MS), as described below. For preliminary PK analyses, a second group of CD-1 mice were injected r.o. with *Ru1* (0.14 mg/kg) and at the indicated time points, blood was collected in EDTA tubes (Aquisel, Cat no. 107545) and analyzed by ICP-MS, as described below.

Indirect calorimetry analyses were carried out using a 16-chamber TSE PhenoMaster monitoring system (TSE Systems GmbH, Bad Homburg, Germany). Full access to food and water was continuously available, and their intake was monitored using built-in devices located within each cage. Calorimetry measurements were carried out during a period of 72 h, according to animal weight, to exclude changes in body weight that would contribute to differences in energy expenditure measurements [28]. Seven days prior to introducing mice into the PhenoMaster monitoring system, 10-week-old C57Bl6 mice (Janvier, France) were subcutaneously implanted (in their back), with Micro-Osmotic Pumps (Azlet® model 1002, which release 0.25µl/hour over the course of 14 days) containing 100µl of 5mM *Ru1* or physiological saline (i.e., Sham). Mice were introduced into individual chambers and were on a 12-hour light-dark cycle (lights on at 07:00am) during the course of the experiment, with a maintained room temperature of 22 ± 2˚C. Oxygen consumption and CO2 release was measured. From these values, respiratory exchange ratio (RER) was determined as VCO2/VO2 and energy expenditure (EE) was calculated as = (3.185+ 1.232 x RER) x VO2.

For *in vivo* tumor growth and Limiting Dilution Analysis (LDA) assays with PDAC cells, female 6- to 8-week-old NU-Foxn1nu nude mice (Janvier, France) were injected subcutaneously with dilutions of *Ru1*-treated (100µM for 24h) or untreated PDAC cells in 50µl Matrigel (Corning) per injection. Tumor growth was monitored bi-weekly for up to 4 months. Mice were sacrificed and tumors were weighed, photographed, and part of each tumor was fixed in 4% PFA and processed for histological analysis. CSC frequencies were calculated using the ELDA software https://bioinf.wehi.edu.au/software/elda/.

For PDX *in vivo* treatment experiments, tumors were initially established by subcutaneously implanting (with Matrigel (Corning)) tumor pieces of the indicated PDXs in the right and left flanks of 6- to 8-week-old NU-Foxn1nu nude mice (Janvier, France). 4-5 weeks post implantation, tumors were excised, cut into identical pieces of approximately 50mm^3^ and implanted (with Matrigel (Corning)) subcutaneously into the left and right flanks of 6- to 8-week-old NU-Foxn1nu nude mice (Janvier, France). Three weeks later, tumors were measured to ensure volumes of 125-150 mm^3^, mice were weighed to calculate treatment concentrations per Kg, randomized into treatment groups (5-6 mice per group) and treatments were initiated for approx. three consecutive weeks. *Ru1* was resuspended in physiological saline (0.45% NaCl) to a concentration of approximately 0.5mM such that mice were treated with a volume of *Ru1* equivalent to 1.4mg/kg. Initially three routes of administration for *Ru1* were tested: 1) orally (100µl daily), 2) via retro-orbital injection (100µl daily) or 3) subcutaneously into the tumor (100µl twice per week). Gemcitabine (Accord Healthcare, S.L.U.) was administered twice a week (50 mg/kg i.p.) and 5FU (Sigma) was administered twice a week (30 mg/kg i.p.). Tumor volumes were determined twice per week by caliper measurements. At the time of sacrifice, mice were weighed, blood was collected in EDTA tubes (Aquisel, Cat no. 107545) and tumors and organs were excised and weighed, photographed, fixed in 4% PFA and processed for histological analysis or analyzed by inductively coupled plasma mass spectrometry (ICP-MS), as described below.

### RNA sequencing analysis

Total RNA was isolated by the guanidine thiocyanate (GTC; VWR AMRESCO Chemicals, Cat no. K965-250ML) method using standard protocols [29]. PolyA+ RNA fraction was processed as in Illumina’s ‘‘TruSeq RNA Sample Preparation v2 Protocol’’. The resulting purified cDNA library was applied to an Illumina flow cell for cluster generation (TruSeq cluster generation kit v5) and sequenced on the Genome Analyzer IIx with SBS TruSeq v5 reagents by following manufacturer’s protocols. RNA-seq data sets were analyzed using the tool Nextpresso [30]. Nextpresso is comprised of four basic levels: 1. Quality check, 2. Read cleaning and/or down-sampling, 3. Alignment, and 4. Analysis (gene / isoform expression quantification, differential expression, gene set enrichment analysis and fusion prediction. Gene signatures (Hallmark gene sets) were downloaded from GSEA - Molecular Signature Database for Gene set enrichment analysis. Data deposited in the NCBI SRA database (Accession: PRJNA832709).

### RNA Preparation and Real-Time PCR

Total RNA from human PDX-derived cell lines, PDX tumors or mouse organs was isolated by the GTC method using standard protocols [29]. One microgram of purified RNA was used for cDNA synthesis using the Thermo Scientific Maxima First Strand cDNA Synthesis Kit (ThermoFisher Scientific) according to manufacturer´s instructions, followed by SYBR green RTqPCR (PowerUp™ SYBR™ Green Master Mix, ThermoFisher Scientific) using an Applied Biosystems StepOnePlus™ real-time thermocycler (ThermoFisher Scientific). Thermal cycling consisted of an initial 10 min denaturation step at 95°C followed by 40 cycles of denaturation (15 sec at 95°C) and annealing/extension (1 min at 60°C). mRNA copy numbers were determined relative to standard curves comprised of serial dilutions of plasmids containing the target coding sequences and normalized to ß-actin levels. Primers used are listed in Supplementary Table S2.

### Probabilistic graphical models

From FPKM data from PDX models, control or treated with *Ru1* or *Ru1*-met, the 2,000 most variable genes were selected. These genes were used to build a probabilistic graphical model without other a priori information based on correlation as associative measurement. Probabilistic graphical model was constructed using R v3.2.5 and *grapHD* package [31]. The result is an undirected graph with local minimum Bayesian Information Criterion (BIC) based on the subsequent steps: first, the spanning tree with the maximum likehood is found and, second, the graph is customized by the adding of edges that reduce BIC and preserve the decomposability [32]. The resulting network was analyzed searching for a functional structure as previously described [33]. Gene ontology analyses were performed using DAVID webtool [34], selecting “homo sapiens” as background and KEGG, Biocarta and GOTERM-FAT as categories. Functional node activities were calculated as the mean expression of the genes included in one functional node related to its overrepresented function. Differences between conditions were assessed by a non-parametric Kruskal-Wallis test using Graph Pad v6.

### Metabolic pathway analyses

Flux Balance Analysis (FBA) is a method to model metabolic networks and to estimate tumor growth [35]. For FBA, the whole human metabolic reconstruction Recon3D [36] and COBRA Toolbox library [37] were used. Recon3D contains information about 10,600 metabolic reactions, 5,835 metabolites and 5,939 Gene-Protein-Reaction rules (GPRs) which contain information about what genes are involved in each metabolic reaction as Boolean expressions. GPRs were solved as described in previous studies [38, 39] using a modification of Barker et al. algorithm [40] and incorporated into the model using a modified E-flux algorithm [39, 41]. Briefly, “OR” operators were solved as the sum and “AND” operators were solved as the minimum. Then, GPRs were normalized using a Max-min function to an interval [0,1] and introduced into the model as the reaction bounds. As objective function the biomass reaction included in the Recon3D was used, as representative of tumor growth. The 10,600 reactions are grouped into 103 metabolic pathways. The mathematical problem was solved using linear programming. To compare metabolic activity between conditions, flux activities were calculated as the sum of fluxes of the reactions included in a concrete metabolic pathway defined in Recon3D. Then, a delta was calculated subtracting *Ru1* to control flux activity for each metabolic pathway.

### Oxygen Consumption Rate (OCR) measurements

Sphere-derived Panc185, PancA6L and Panc215 cells were plated in XF HS Miniplates (Seahorse Bioscience) at a cellular density of 5,000 cells/well. For OCR determination, cells were incubated in Seahorse XF DMEM media (103680, Agilent) supplemented with 2mM glutamine, 10mM glucose, and 1mM pyruvate for 1 h, prior to the measurements using the Seahorse XFp Cell Mito Stress Kit (103010, Agilent). After an OCR baseline measurement, the minimum oxygen consumption was determined adding 1.5µM oligomycin (O) and the maximal respiration rate was assessed by adding 1µM FCCP (F). At the end of the experiment the non-mitochondrial oxygen consumption was evaluated adding both 0.5µM rotenone (R) and antimycin (A). Experiments were run in a XF HS Mini analyzer (Seahorse Agilent), and raw data were normalized to total protein using BCA protein assay kit (Cat. no. 23225, Thermo Scientific).

### Lactate production assay

Supernatant from indicated cells was collected to evaluate the changes in the levels of lactate production. The analysis was performed using the Lactate Assay Kit (Sigma-Aldrich, St. Louis, Missouri, USA) according to the manufacturer’s instructions. The optical density was determined using a Synergy™ HT Multi-Mode Microplate Reader (BioTek, Winooski, Vermont, USA) at a wavelength set to 570nm. Data were normalized to total protein using the BCA protein assay kit (Thermo Scientific).

### ATP determination assay

Lysate pellets of cells from control and treated cells were collected to evaluate the changes in the levels of ATP. The analysis was performed using the ATP Bioluminiscense Assay Kit CLS II (Cat. no. 11699695001, Roche) according to the manufacturer’s instructions. Bioluminiscence was determined using a Synergy™ HT Multi-Mode Microplate Reader (BioTek, Winooski, Vermont, USA). Data were normalized to total protein using the BCA protein assay kit (Thermo Scientific).

### Immunostainings and confocal analysis

For fluorescence confocal microscopy, indicated cells were seeded in 8-well IbiTreat (ibidi chamber slides, Cat no. 181009/1) in RPMI (Gibco) containing 10% FBS (Thermo Fisher Scientific) at 37°C, 5% CO2. After indicated treatments with *Ru1* or *Ru-TMR* and indicated time points, the medium was removed, and cells were stained with Mitotracker Green (MTR-G, M7514, Invitrogen) at a final concentration of 20nM in serum-free RPMI for 30 min at 37°C, washed with PBS, and then overlaid with fresh RPMI containing 10% FBS. The fluorescent images were collected immediately afterwards with a laser scanning confocal microscope Zeiss 710 40X Apochromatic and analyzed using the software ZEN 2009. For fluorescence confocal microscopy of Tetramethylrhodamine, ethyl ester, perchlorate (TMRE) and CellROX DeepRed, indicated cells growing in glass-bottom cell culture plates were treated with 100µM *Ru1* or *Ru1-met*. After 24 hours of incubation, cells were washed twice with RPMI containing 10% FBS. 1µM TMRE (Cat no. T669, ThermoFisher Scientific) or 10 µM CellROX-DR (Cat no. C10422, ThermoFisher Scientific) reagent in RPMI containing 10% FBS were added for 20 or 30 min, respectively. Then, two new washes with RPMI containing 10% FBS were performed, and cells were observed in an Andor Dragonfly Spinning Disc confocal system attached to a Nikon Eclipse TiE using a 60X apochromatic objective and adequate filter settings.

### Electron microscopy analysis

PaCSC-enriched spheres were trypsinized and centrifuged for 5 min at 400×g. Cell pellets were fixed using a solution of 2.5% glutaraldehyde in cacodylate buffer 0.1M for 60 min. Cell pellets were post-fixed in osmium tetroxide, dehydrated through ascending concentrations of ethanol and embedded in epoxy resin. Ultra-thin sections were obtained at 0.1μm, counterstained with uranyl acetate and lead citrate prior to image acquisition with a JEOL JEM1010 (100 kV) transmission electron microscope equipped with a Gatan Orius 200 SC camera. Images were processed using DigitalMicrograph (Gatan, Inc).

### Western blot analysis

Cells were harvested in RIPA buffer (Sigma-Aldrich) supplemented with a protease inhibitor cocktail (Roche Applied Science, Indianapolis, IN). Fifty micrograms of protein were resolved by SDS-PAGE and transferred to PVDF membranes (Amersham Pharmacia, Piscataway, NJ). Membranes were sequentially blocked with 1X TBS containing 5% BSA (w/v) and 0.5% Tween20 (v/v), incubated with a 1:500-1:1000 dilution of indicated antibodies (see Supplementary Table S1) overnight at 4ºC, washed 5 times with 1X TBS containing 0.5% Tween20 (v/v), incubated with horseradish peroxidase-conjugated goat anti-rabbit or goat anti-mouse antibody (Amersham), and washed again to remove unbound antibody. Bound antibody complexes were detected with SuperSignal chemiluminescent substrate (Amersham) and images were obtained using MyECL Imager (Thermo Fisher Scientific). Densitometry histograms were obtained by measuring the intensity of the bands and normalized by their housekeeping loading control by ImageJ software. Blots are accompanied by the locations of molecular weight/size markers (M_r_(K)), as determined using commercially available protein ladders (Novex®Sharp Pre-Stained Protein Ladder Cat no. LC5800 or PageRuler™ Prestained Protein Ladders Cat nos. 26616 or 26619, all from ThermoFisher Scientific).

### Mitochondrial gradient purification

Enrichment of mitochondria prior to density gradient purification was performed following the protocol by Fernández-Vizarra *et al.,* [42]. Briefly, 8×10^7^ cells (untreated or treated for 24 h with 100µM *Ru1*) were mechanically broken in IB buffer [35mM Tris-HCL pH 7.8; 5mM MgCl_2_ (v/v); 25mM NaCl (v/v)] prior to density gradient isolation following the protocol described by Frezza C. *et al.,* [43]. Gradient isolated mitochondria were then resuspended in IBC buffer [0.1M Tris-MOPS; 2mM MgCl_2_ (v/v); 0.2M Sucrose (v/v)] pH 7.4.

Three downstream analyses were performed with purified mitochondria. 1) To determine the amount of *Ru1* in purified mitochondria isolated from control- and *Ru1*-treated cells, density gradient-purified mitochondria were diluted with Nitric Acid (HNO_3_) to a final concentration of 60% for ICP-MS analysis, as described below. 2) To determine the capacity of *Ru1* to enter directly into mitochondria and interact with mtDNA, density gradient-purified mitochondria from untreated cells were incubated for 2 h with 100µM *Ru1*, 100µM *Ru1-met* or an equal volume of H_2_0 (diluent), all in IBC buffer. Before incubation, 0.1µg/µl of DNAse (Cat no. DN25, Sigma-Aldrich) was added to the purified mitochondria to avoid binding of Ru compounds to mtDNA from broken mitochondria. After incubation of purified mitochondria with Ru compounds, mitochondria were resuspended with an equal volume of saturated phenol (pH 8) for subsequent DNA extraction and ICP-MS analysis, both as described below. 3) To determine the capacity of *Ru1* to directly interact with mtDNA, phenol-chloroform-extracted mtDNA from density gradient-purified mitochondria isolated from untreated cells were treated for 1h with diluent or 10µM of *Ru1* or *Ru1-met* prior to PCR, as described below.

### DNA extraction and PCR

DNA was isolated using standard phenol-chloroform extraction methods. For PCR amplification of the D-loop or *RNR2* regions of the mtDNA, 0.1 or 0.01ng of untreated or treated purified mtDNA was used as a template with primers specific to the two aforementioned regions (Supplementary Table S3). Thermal cycling, using a SimpliAmp (ThermoFisher Scientific), consisted of an initial 7 min denaturation step at 95°C, 35 cycles of denaturation (30 sec at 95°C), annealing/extension (1 min at 54°C) and elongation (2 min at 72°C), and a final elongation of 10 min at 72°C. PCR products were resolved on a 1% Agarose/TBE gel for 1 h at 100V, and size verification was performed comparing SybrSafe-stained experimental bands to molecular weight bands of the 1kb Plus DNA Ladder (Cat no. 12308-011 Invitrogen, ThermoFisher Scientific).

### Inductively coupled plasma mass spectrometry (ICP-MS)

To quantify the presence of ruthenium, mtDNA extracted from untreated or *Ru1*-treated mitochondria or cell pellets were dissolved in 100μl of 65% nitric acid in water and then analyzed by ICP-MS. For ICP-MS analysis of extracted PDX354 tumors and/or organs (liver, kidney and brain), weighed tissue samples were cut into small pieces and homogenized in a 15mL falcon tube. Sixty-five percent nitric acid was added to completely cover the tissue, and the mixture was digested overnight. After digestion, water was added to 3 mL, and the solution was centrifuged at 3000-4000 X g at 4°C for 10 min. The pellet was discarded, and the supernatant transferred to an Eppendorf tube for ICP-MS analysis. For serum samples, approximately 30-50µl of serum was dissolved in 65% nitric acid, and water was added to 3 mL for ICP-MS analysis.

ICP-Mass was performed at the CACTUS-Campus Lugo facility of the University of Santiago de Compostela using an ICP-MS Agilent 7700x with a Peltier (2°C) cooled sample introduction system based on a glass low-flow MicroMist Nebulizer and a quartz torch double pass spray chamber for aerosol filtering. The Ru calibration standards were prepared from a 1g/L commercial standard (Merck). Ir (Merck) was used as an internal standard.

### Statistical analyses

Results are presented as means ± standard error of the mean (sem) unless stated otherwise. Pair-wise multiple comparisons were performed with one-way ANOVA (two-sided) with Bonferroni or Dunnett adjustment, as indicated in the figure legends. Student’s t-test were used to determine differences between means of groups. *P* values <0.05 were considered statistically significant. All analyses were performed using GraphPad Prism version 6.0c (San Diego California USA).

## Results

### Ru1 affects the molecular and functional properties of PaCSCs

**Fig. 1:**
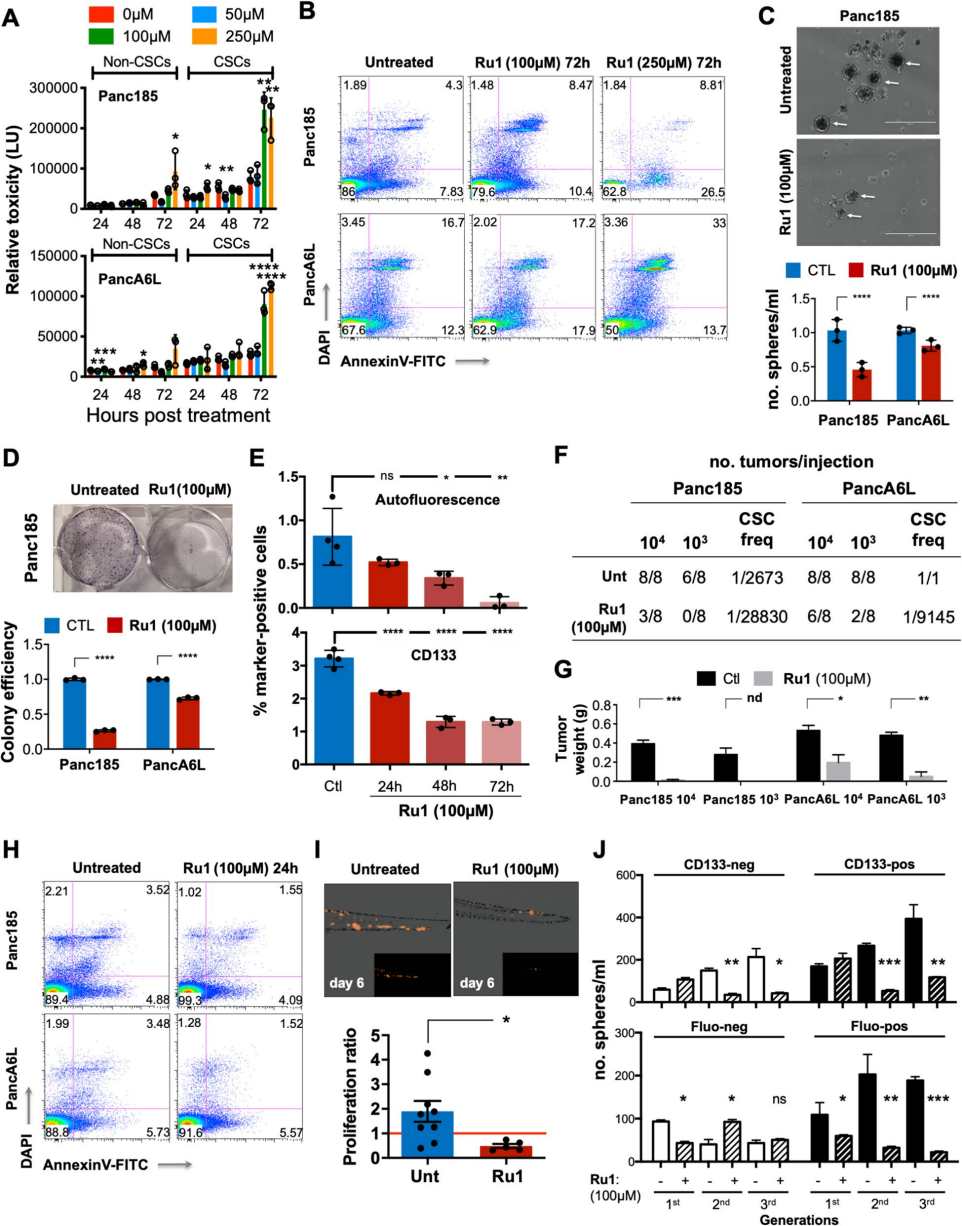
*Ru1 negatively affects PaCSCs molecular and functional properties.* **A** Relative toxicity (LU = light units) ± SD in Panc185 and PancA6L adherent (non-CSCs) and sphere (CSCs) cultures treated at the indicated doses of *Ru1* for 24, 48 or 72 h. Toxicity was determined using the ToxiLight assay kit at the indicated hours post treatment. **B** Representative flow cytometric plots of AnnexinV-FITC staining in Panc185 and PancA6L sphere-derived cells treated for 72 h with *Ru1* at 100 and 250µM. **C** Top: Representative images of Panc185 spheres in the absence (untreated) or presence of *Ru1*. Cells were treated with *Ru1* for 24 h at 100µM prior to establishing spheres. Bottom: Mean fold change ± SD in the number (no.) of spheres formed compared to control (set as 1.0). **** *p* < 0.0001, as determined by unpaired two-sided Student’s t-test. **D** Top: Representative images of Panc185 colonies in the absence (untreated) or presence of *Ru1*. Cells were treated with 24 h with the *Ru1* at 100µM during day 1 post seeding. Bottom: Mean fold change ± SD in the colony efficiency in untreated and *Ru1*-treated samples, compared to control (set as 1.0). **** *p* < 0.0001, as determined by unpaired two-sided Student’s t-test. **E** Mean percentage of Autofluorescent+ or CD133+ cells ± SD, determined by flow cytometry, in untreated and *Ru1*-treated Panc185 cells (* *p* <0.05, ** *p* < 0.01, **** *p* < 0.0001, ns = not significant, as determined by unpaired Student’s t test). **F** Sum total of tumors obtained from untreated or *Ru1*-treated Panc185 and PancA6L injections, from two independent Limiting Dilution Analysis (LDA) assays. Cells were treated with *Ru1* for 24h at 100µM prior to injection. CSC frequencies were calculated using the ELDA software. **G** Average tumor weights ± SD. * *p* < 0.05, ** *p* < 0.01, *** *p* < 0.001, as determined by unpaired two-sided Student’s t-test; nd= not determined. **H** Representative flow cytometric plots of AnnexinV-FITC/DAPI staining in Panc185 and PancA6L cells, treated for 24 h with *Ru1* at 100 µM, and subsequently injected *in vivo*. **I** Top: Representative images of zebrafish embryo tails at 6 days post injection of 100-150 untreated or *Ru1*-treated H2b-mCherry-labelled Panc185 cells. Scale bar = 250 μM. Bottom: Mean ± SEM of proliferation ratio observed between untreated (Unt) or *Ru1*-treated determined on day 6 post injection. Cells were treated with *Ru1* for 24 h at 100 µM prior to injection. Proliferation ratios are represented in comparison to 1 dpi (day post injection) (red line). * *p* < 0.05, as determined by unpaired two-sided Student’s t-test. **J** Mean number (no.) of spheres ± SEM determined at 7 (1^st^ generation), 14 (2^nd^ generation), and 21 (3^rd^ generation) days post seeding, from CD133-negative, CD133-positive, Autofluorescent (Fluo)-negative, or Fluo-positive cells sorted from Panc185 cells and treated at d0 with *Ru1* (100µM) for 24h. * *p* < 0.05, ** *p* < 0.01, *** *p* < 0.001, as determined by unpaired two-sided Student’s t-test

Since the ruthenium complex [Ru(terpy)(bpy)Cl]Cl (*Ru0*) undergoes a relatively rapid aquation in aqueous buffers to produce *Ru1* ([Ru(terpy)(bpy)H_2_O]^+2^Cl^-2^) [15, 44] (Fig. S1A-C), we decided to directly use the aquo derivative for this study. The required chloride-water exchange can be accelerated by irradiation with visible light for 60-120 min (Fig. S1D). We have previously shown that *Ru1* presents very low *in vitro* toxicity in HeLa cells [15], results that are in concordance with previous studies using *Ru0* [45]. Nonetheless, we tested the toxicity of *Ru1* in two primary PDX-derived PDAC cultures grown as adherent 2D monolayers (non-CSC-enriched) or as 3D spheres (CSC-enriched), using a sensitive luminescence-based toxicity assay. Minimal cytotoxicity 24 and 48 h post continuous treatment at all doses tested was observed (Fig. 1A). However, we did observe toxicity after 72 h of continuous treatment at 100 and 250µM, with a more potent effect observed in CSC-enriched cultures (Fig. 1A). This selective effect in 3D sphere (CSC-enriched) cultures at 72 h with 250µM *Ru1* was due to induced apoptosis (Fig. 1B). With this information in hand, we tested the effect of *Ru1* on CSC self-renewal *in vitro* by assessing sphere and colony formation capacity following a single 24 h treatment with a non-toxic/non-apoptotic-inducing dose of *Ru1* (100µM) (Fig. 1C-D). Concomitant with reduced self-renewal and clonogenicity, we observed a time dependent reduction in established PaCSC markers in Panc185 cells, namely autofluorescence [46] and CD133 [47] (Fig. 1E).

This apparent reduction in the CSC compartment was functionally validated *in vivo* in a limiting dilution assay (LDA) with cells pre-treated 24 h with *Ru1* prior to injection, revealing impaired tumorigenic potential and decreased CSC frequencies and tumor weights (Fig. 1F-G). Importantly, cells were confirmed to be non-apoptotic prior to injection (Fig. 1H). To evaluate tumor formation *in vivo* at earlier times, we used the zebrafish xenograft model, which showed that *Ru1*-pre-treated cells gave rise to significantly fewer microtumors at day 6 post-injection (p.i.) (Fig. 1I), when compared with control cells. Finally, to confirm the specificity of *Ru1* on the CSC compartment, CD133- and autofluorescent-negative and positive cells were sorted and placed in 3D sphere conditions in the presence or absence of the compound. As expected, the marker-positive populations had a greater self-renewal (i.e., sphere forming) capacity over several generations, but were at the same time more sensitive to *Ru1* compared to the marker-negative sorted populations, particularly when using autofluorescence to separate CSCs from non-CSCs (Fig. 1J).

### Ru1 halts PDX growth *in vivo*

**Fig. 2:**
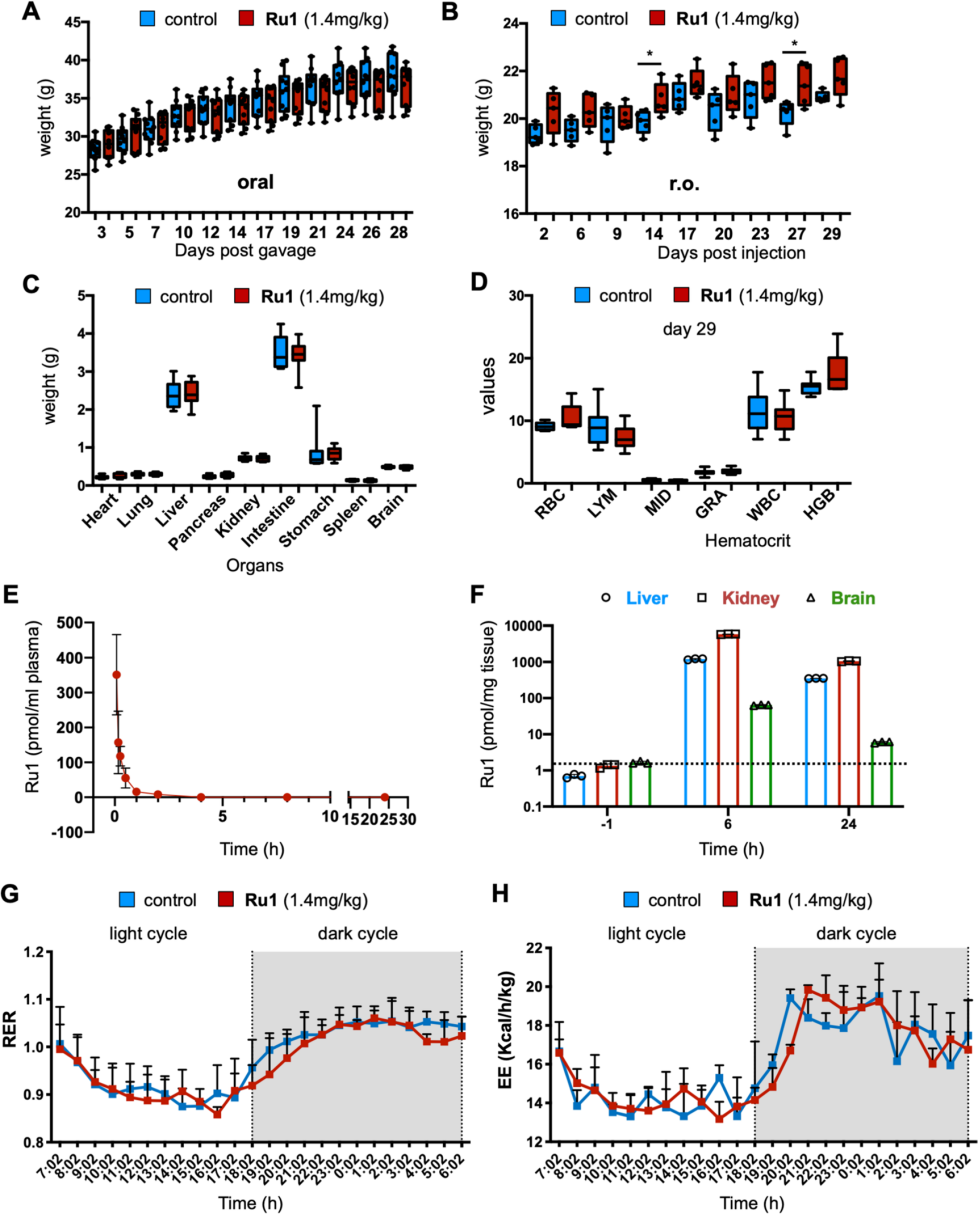
*Analysis of Ru1 toxicity, PK and distribution in vivo.* **A-B** Average weight ± SEM of mice treated orally (**A**) or retro-orbitally (r.o.) (**B**) with diluent control (Ctl) or *Ru1* (1.4mg/kg,) for approximately 28-29 days. (*p < 0.05, as determined by unpaired two-sided Student’s t-test). **C** Average weight ± SEM of indicated organs extracted on d29 from mice treated with diluent control (Ctl) or *Ru1* (1.4mg/kg, r.o). No significant differences were found, as determined by unpaired two-sided Student’s t-test. **D** Average values ± SEM of indicated hematocrit parameters determined from blood of mice extracted on d29 post treatment with diluent control (Ctl) or *Ru1* (1.4mg/kg, r.o). No significant differences were found, as determined by unpaired two-sided Student’s t-test. **E** Picomoles of *Ru1* per ml of serum, determined by ICP-MS, from mice at indicated time points post treatment initiation. **F** Picomoles of *Ru1* per mg of tissue, determined by analyzing ruthenium with ICP-MS, from liver, kidneys and brain, extracted at indicated time points post treatment initiation. Dashed line indicates the background of the assay. **G-H** Indirect calorimetry analyses of mice treated with Ru1. Respiratory exchange ratio (RER) was determined as VCO2/VO2 and Energy expenditure (EE) was calculated as (3.185+ 1.232 x RER) x VO2. Shown are the mean RER (**G**) and mean EE (**H**) values ± SD for mice implanted with Azlet® Micro-Osmotic Pumps containing 5mM Ru1 or physiological saline (i.e., Sham) as a function of time (24 hours)

Based on these promising results, we next investigated the effects of *Ru1* *in vivo*. First, CD-1 mice were treated with a maximum feasible dose of *Ru1* (1.4mg/kg), administered via oral gavage (o.g., 100µl daily) or retro-orbital (r.o.) injection (100µl twice per week), and mice were weighed at the indicated times post treatment initiation. *Ru1* treatment had no effect on body weight over the course of 4 weeks (Fig. 2A-B), nor on organ weight or serological parameters at the conclusion of the experiment (Fig. 2C-D). Importantly, no adverse effects on serological parameters (Fig. S2A-C) nor on subtle neurological perturbations (as determined by Irwin’s test) were observed at earlier acute time points. Preliminary pharmacokinetics (PK) and biodistribution studies were performed to better understand the distribution and metabolism of the *Ru1* in vivo. Ruthenium was detected in blood, by ICP-MS, at 5 min post injection, and levels quickly declined at subsequent time points analyzed suggesting that *Ru1* has a short but acceptable ½ life in vivo (Fig. 2E). Indeed, a preliminary biodistribution analysis indicated that the majority of Ru1 is metabolized by the liver and is likely cleared by the kidneys (Fig. 2F). Lastly, an indirect calorimetry analyses was performed, and no differences were observed between treated and untreated mice at the level of Respiratory Exchange Rate (RER) and Energy Expenditure (EE) (Fig. 2G-H), illustrating that, under these conditions, *Ru1* is non-toxic *in vivo*.

**Fig. 3:**
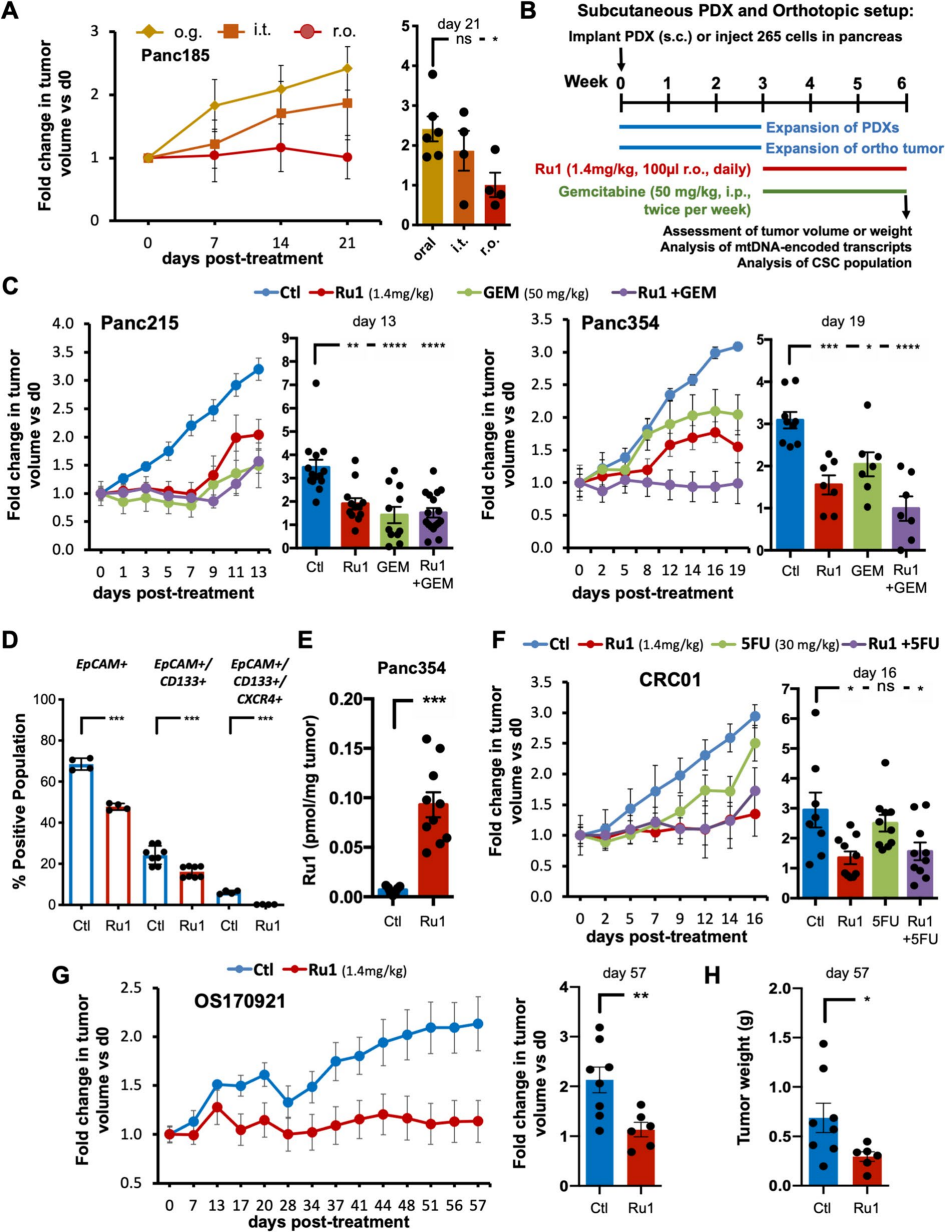
*Ru1 halts PDAC, CRC and OS PDX growth in vivo.* **A** Left: Average fold change in tumor volume ± SEM in mice bearing Panc185 PDXs and treated with *Ru1* orally (o.g.), intra tumoral (i.t.) or retro orbitally (r.o.) over the course of 21 days and compared to d0 (*n*=4-6 tumors/group). Right: Fold change in tumor volume ± SEM determined on day 21 post treatment initiation. *p < 0.05; ns= not significant, as determined by one-way ANOVA with Dunnett post-test, compared to oral. **B** Experimental set-up for *in vivo* experiments using subcutaneously or orthotopically implanted PDXs and treatment with *Ru1* (1.4 mg/kg; daily) and/or Gemcitabine (50mg/kg; twice per week). **C** Average fold change in tumor volume ± SEM in mice bearing Panc215 PDXs (left) or Panc354 PDXs (right) and treated with diluent control (Ctl), *Ru1* (1.4mg/kg r.o.; daily), Gemcitabine (50mg/kg; twice per week) or a combination of both and compared to d0 (*n*=6-8 tumors/group). Histograms: Fold change in tumor volume ± SEM determined at treatment cessation. *p < 0.05, **p < 0.01, *** *p*<0.001, **** *p*<0.0001, as determined by one-way ANOVA with Dunnett post-test, compared to Ctl. **D** Mean percentage of EpCAM+, EpCAM+/CD133+, or EpCAM+/CD133+/CXCR4+ PaCSCs ± SEM, determined by flow cytometry, in extracted Panc354 tumors from (C). *** *p*<0.001, as determined by unpaired two-sided Student’s t-test. **E** Picomoles of *Ru1* per mg of tumor, determined by ICP-MS, from PDX354 tumors extracted on d19 post treatment initiation (approx. 30-32h after the final injection). **F** Left: Average fold change in tumor volume ± SEM in mice bearing CRC01 treated with diluent control (Ctl), *Ru1* (1.4mg/kg r.o.; daily), 5FU (30mg/kg; twice per week) or a combination of both and compared to d0 (*n*=8-12 tumors/group). Right: Fold change in tumor volume ± SEM determined at treatment cessation (d16). *p < 0.05; ns= not significant, as determined by one-way ANOVA with Dunnett post-test, compared to Ctl. **G** Left: Average fold change in tumor volume ± SEM in mice bearing OS170921 treated with diluent control (Ctl) or *Ru1* (daily 1.4mg/kg r.o) and compared to d0 (*n*=6-8 tumors/group). Right: Fold change in tumor volume ± SEM determined at treatment cessation (d57). **p < 0.01, as determined by unpaired two-sided Student’s t-test. **H** OS170921 tumor weights (g) ± SEM determined at treatment cessation (d57). *p < 0.05, as determined by unpaired two-sided Student’s t-test

To determine the optimal administration route that could lead to an anti-tumor effect, avatar mice with subcutaneously implanted PDAC PDXs of Panc185 were randomized and administered *Ru1*: 1) o.g. (1.4mg/kg, daily), 2) r.o. (1.4mg/kg, daily) or 3) intra-tumoral (i.t.) (100µl of 500µM, twice per week). Monitorization of tumor volume by caliper measurement revealed that while o.g. and i.t. administration had no effect on tumor growth, r.o. administration was cytostatic, resulting in growth inhibition (Fig. 3A). Moreover, we confirmed that the ruthenium complex reaches the tumor by ICP-MS (Fig. S2D). Next, we subcutaneously implanted PDXs of 2 PDAC tumors and further tested the efficacy of *Ru1* alone and in combination with gemcitabine, via r.o. administration (Fig. 3B-C). *Ru1* at 1.4mg per kg body weight, halted PDX354 and PDX215 tumor growth (Fig. 3C). When combined with gemcitabine, an additive effect was observed for PDX354 (Fig. 3C). Moreover, we observed a reduction in the percentage of epithelial (i.e., EpCAM+) cells within the tumor, and a significant decrease in the percentages of CD133+ and CD133+/CXCR4+ cells within the EpCAM+ cell population, which is in line with our hypothesis that *Ru1* affects the CSC compartment (Fig. 3D). Importantly, ICP-MS analysis confirmed the presence of ruthenium in *Ru1*-treated tumors (Fig. 3E). Since colorectal tumor are also driven by CSCs [48], we tested the effect of *Ru1* against a CRC PDX model, alone and in combination with Fluorouracil (5FU), and observed significant tumor growth delay (Fig. 3F). We also tested *Ru1* in a PDX model of osteosarcoma (OS), the most common primary bone tumor in children and adolescents and a tumor where CSCs are believed to drive metastasis and contribute to poor prognosis in advanced OS patients [49]. Again, *Ru1* significantly delayed tumor growth (Fig. 3G-H).

**Fig. 4:**
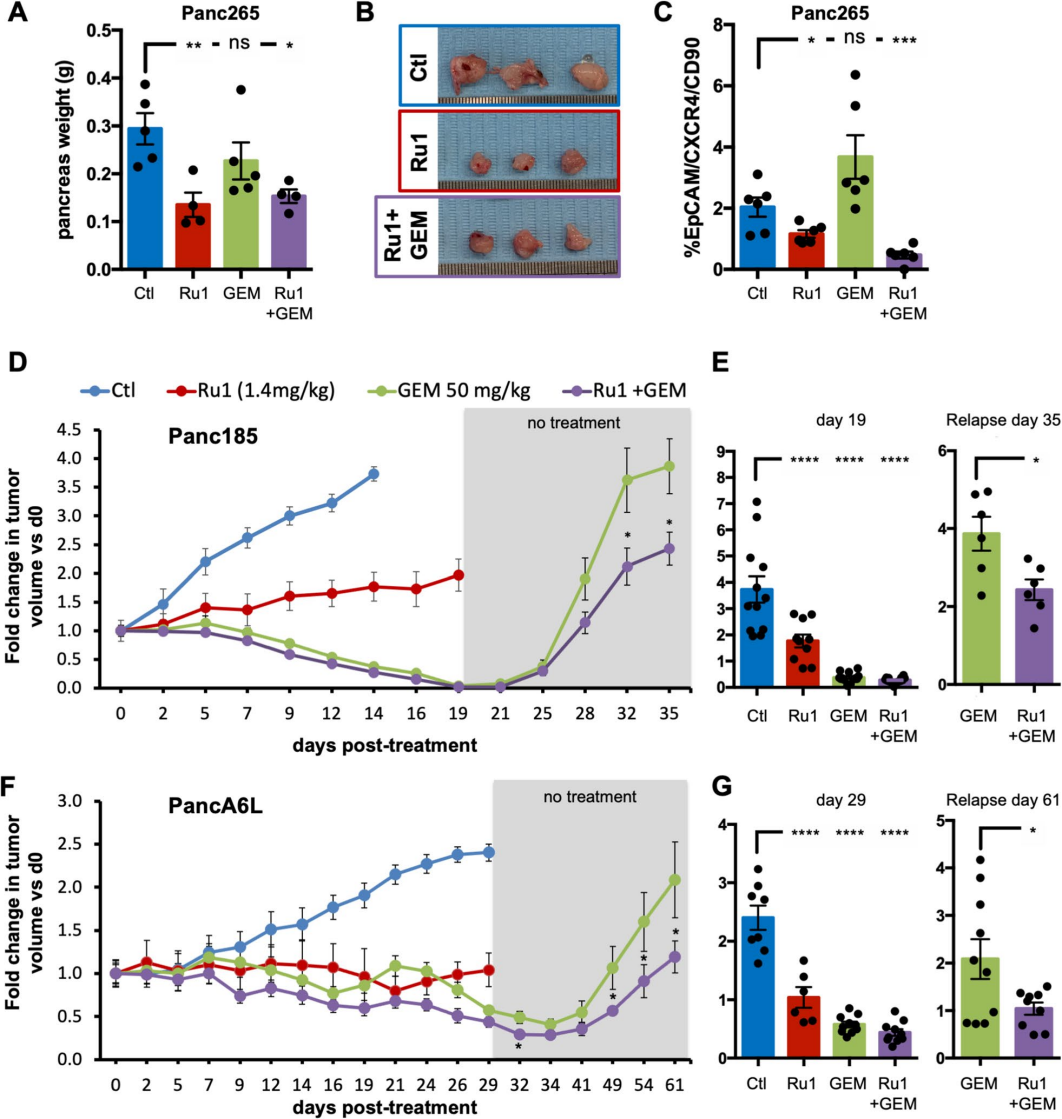
*Ru1 halts PDAC growth and relapse in vivo.* **A** Fold change in pancreas weight ± SEM in mice injected orthotopically with Panc265 cells determined at treatment cessation (*n*=4-5 mice/group). *p < 0.05, ** *p*<0.01, ns = not significant, as determined by one-way ANOVA with Dunnett post-test, compared to Ctl. **B** Representative photos of Panc265 orthotopic tumors extracted from mice 3 weeks post treatment initiation. **C** Mean percentage of EpCAM+/CXCR4+/CD90+ PaCSCs ± SD, determined by flow cytometry, in extracted tumors from (**A**). *p < 0.05, *** *p*<0.001, ns = not significant, as determined by one-way ANOVA with Dunnett post-test, compared to Ctl. **D** Average fold change in tumor volume ± SEM in mice bearing Panc185 PDXs and treated with diluent control (Ctl), *Ru1* (1.4mg/kg r.o.; daily), Gemcitabine (50mg/kg; twice per week) or a combination of both and compared to d0 (*n*=6-12 tumors/group). **E** Fold change in Panc185 tumor volumes ± SEM determined at treatment cessation and at the indicated time during relapse. *p < 0.05; **** *p*<0.0001, as determined by unpaired two-sided Student’s t-test or a one-way ANOVA with Dunnett post-test, compared to Ctl. **F** Average fold change in tumor volume ± SEM in mice bearing PancA6L PDXs and treated with diluent control (Ctl), *Ru1* (1.4mg/kg r.o.; daily), Gemcitabine (50mg/kg; twice per week) or a combination of both and compared to d0 (*n*=6-12 tumors/group). **G** Fold change in PancA6L tumor volumes ± SEM determined at treatment cessation and at the indicated time during relapse. *p < 0.05; **** *p* <0.0001, as determined by unpaired two-sided Student’s t-test or a one-way ANOVA with Dunnett post-test, compared to Ctl

To test *Ru1* in a more physiologically relevant model, mice were orthotopically implanted with PDX Panc265 cells in the pancreas and treated as described (Fig. 3B). Three weeks post treatment, *Ru1* alone and *Ru1* in combination with gemcitabine significantly reduced tumor volumes (Fig. 4A-B). This effect was accompanied again by a reduction in the PaCSC population (EpCAM+/CXCR4+/CD90+), with the combination treatment having the most significant effect (Fig. 4C). Finally, we established two additional PDX models (Panc185 and PancA6L) and measured tumor growth during treatment and after treatment removal. Again, *Ru1* alone was cytostatic and when combined with gemcitabine, an equal or additive effect (compared to gemcitabine alone) was observed (Fig. 4D-G). Interestingly, when treatment was terminated, the kinetics of tumor relapse was significantly lower in *Ru1* plus gemcitabine versus gemcitabine alone groups (Fig. 4D-G), suggesting more effective elimination of the PaCSC population when *Ru1* was used.

### Mechanistic experiments in cell cultures using Ru1 derivatives

**Fig. 5:**
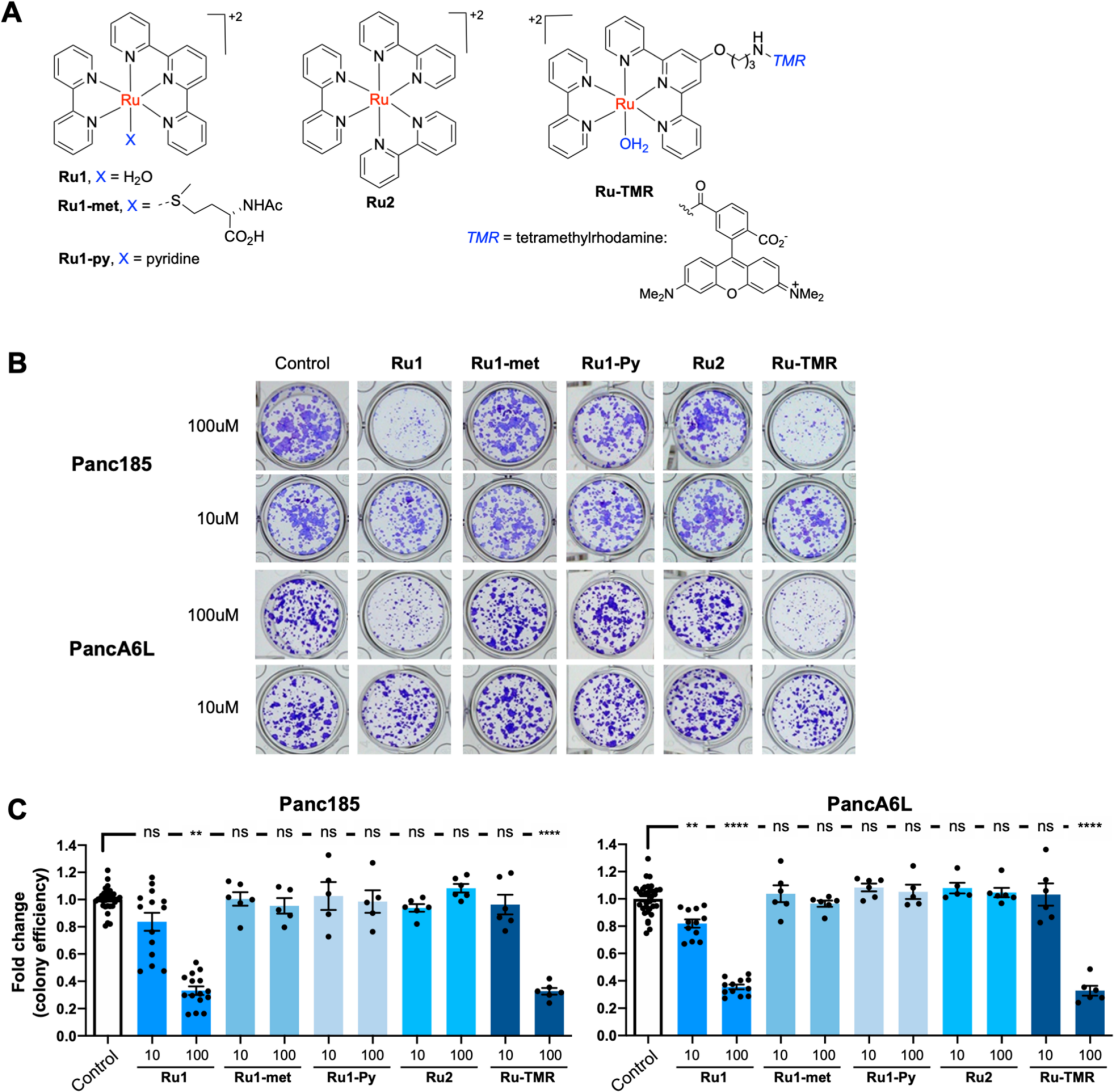
*Control assays using Ru1 derivatives.* **A** Chemical structure of *Ru1* and derivatives. **B** Representative images of Panc185 and PancA6L colonies in the absence (Control) or presence of *Ru1* or indicated derivatives (10 and 100µM). Cells were treated for 24 h with the ruthenium compounds at 10 or 100µM during day 1 post seeding. **C** Mean fold change ± SEM in the colony efficiency (crystal violet absorbance) in treated (10 or 100µM) and Control samples, which were set to 1.0. ** *p* < 0.01, **** *p* < 0.0001, ns = not significant, as determined by one-way ANOVA with Dunnett post-test, comparing 10- or 100µM-treated to Control

The above data suggest that *Ru1* likely affects key stem properties of PaCSCs. To obtain more information on key molecular parameters that influence this anti-CSC activity, we synthesized selected derivatives that included designed modifications in the metal ligands (Fig. 5A). The activity of these derivatives was assessed using a clonogenicity assay (colony formation assay). The results, summarized in Fig. 5B-C, revealed that only compounds *Ru1* and *Ru-TMR*, which present an exchangeable (aquo) ligand in the coordination sphere, are functionally active at 100µM. The observation that derivatives *Ru1-met*, *Ru2* and *Ru1-Py* (ruthenium complexes in which the sixth coordination position is occupied with a non-labile ligand) are inactive, is clearly indicative of a critical role of the exchangeable (aquo) ligand. Therefore, the molecular mechanism of action may be associated to the metalation of biomolecular targets, likely, accessible guanines in nucleic acids, which is in line with our previous observations in “*in vitro”* experiments using DNA samples [15].

### Ru1 negatively regulates genes involved in OXPHOS

**Fig. 6:**
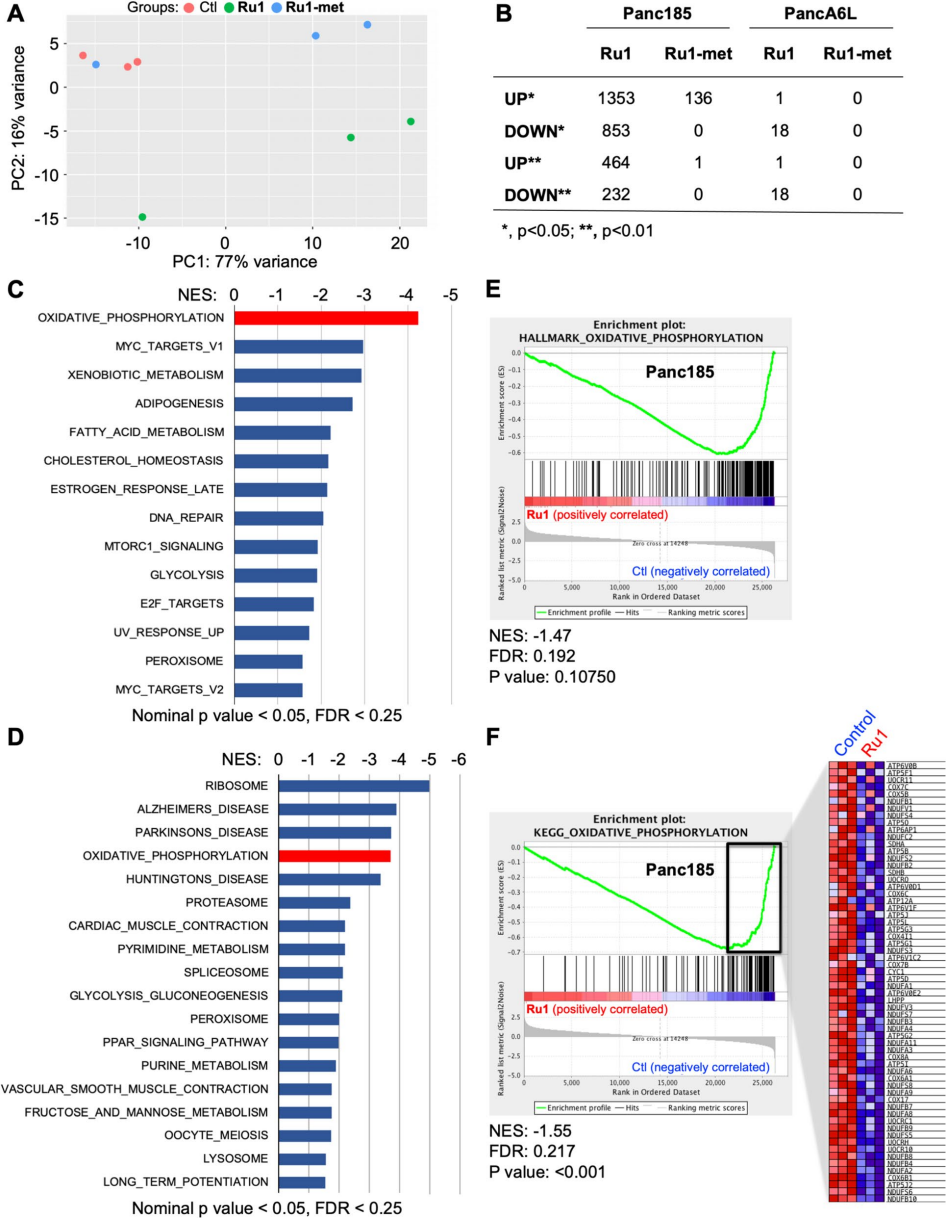
*Ru1 negatively regulates genes involved in OXPHOS.* **A** Principal component (PC) analysis of Control-, *Ru1*- and *Ru1-met*-treated Panc185 spheres (100µM, 24 h). **B** Summary of the number of genes transcriptional modulated (up or down) compared to Control in *Ru1*- and *Ru1-met*-treated Panc185 or PancA6L spheres (100µM, 24 h). **C-D** Gene sets enriched in the transcriptional profile of Panc185 spheres treated with *Ru1* (100µM, 24 h) compared to untreated Controls. Shown are the NES (normalized enrichment score) values for each pathway using the Hallmark or KEGG genesets, with nominal *p* value of <0.05 and FDR < 25%. **E-F** Example enrichment plots for Oxidative Phosphorylation using the Hallmark or KEGG genesets. For (**F**), a heatmap of modulated genes was included

To gain further insight into *Ru1*’s mechanism of action, RNA sequencing (RNAseq) was performed on Panc185 and PancA6L sphere-derived cells treated for 24 h with 100µM of *Ru1* or the unreactive derivative *Ru1-met*. Principle component analysis showed that *Ru1*-treated Panc185 cells clustered separate from control and *Ru1-met*-treated cells (Fig. 6A). Indeed, *Ru1-met* significantly modulated less genes compared to *Ru1* in Panc185 cells and no genes in PancA6L cells (Fig. 6B). Upon increasing statistical significance (*p*<0.01), *Ru1-met* showed no effect on gene modulation in Panc185-treated cells. Interestingly, in *Ru1*-treated PancA6L cells only 1 gene was upregulated (*MT-TF*), whereas 18 genes were down-regulated, all of which were mitochondrial-related genes, 14 exclusively encoded by the mtDNA, and of these 14 genes, 11 are mitochondrial protein coding genes (Fig. S3A). This finding points towards a direct inhibition of mtDNA transcription by the ruthenium complex. Importantly, the same 18 genes downregulated by *Ru1* in PancA6L cells were also downregulated in Panc185 cells, and no downregulation in *POLRMT* nor the two initiation factors *TFAM* and *TFB2M* were observed in Panc185 or PancA6L (Fig. S3B), excluding effects on the mtRNA polymerase as the mechanism underlying the observed downregulation of mtDNA-encoded genes. Normalized Fragments Per Kilobase Million (FPKM) values were processed with Gene Set Enrichment Analysis (GSEA) to identify pathways negatively regulated by *Ru1* in Panc185 cells. Using both the “Hallmark” and “Kegg” genesets collections, we observed several common pathways significantly downregulated (nominal *p*<0.05, FDR<0.25), including several metabolic pathways such as OXPHOS and glycolysis (Fig. 6C-F).

**Fig. 7:**
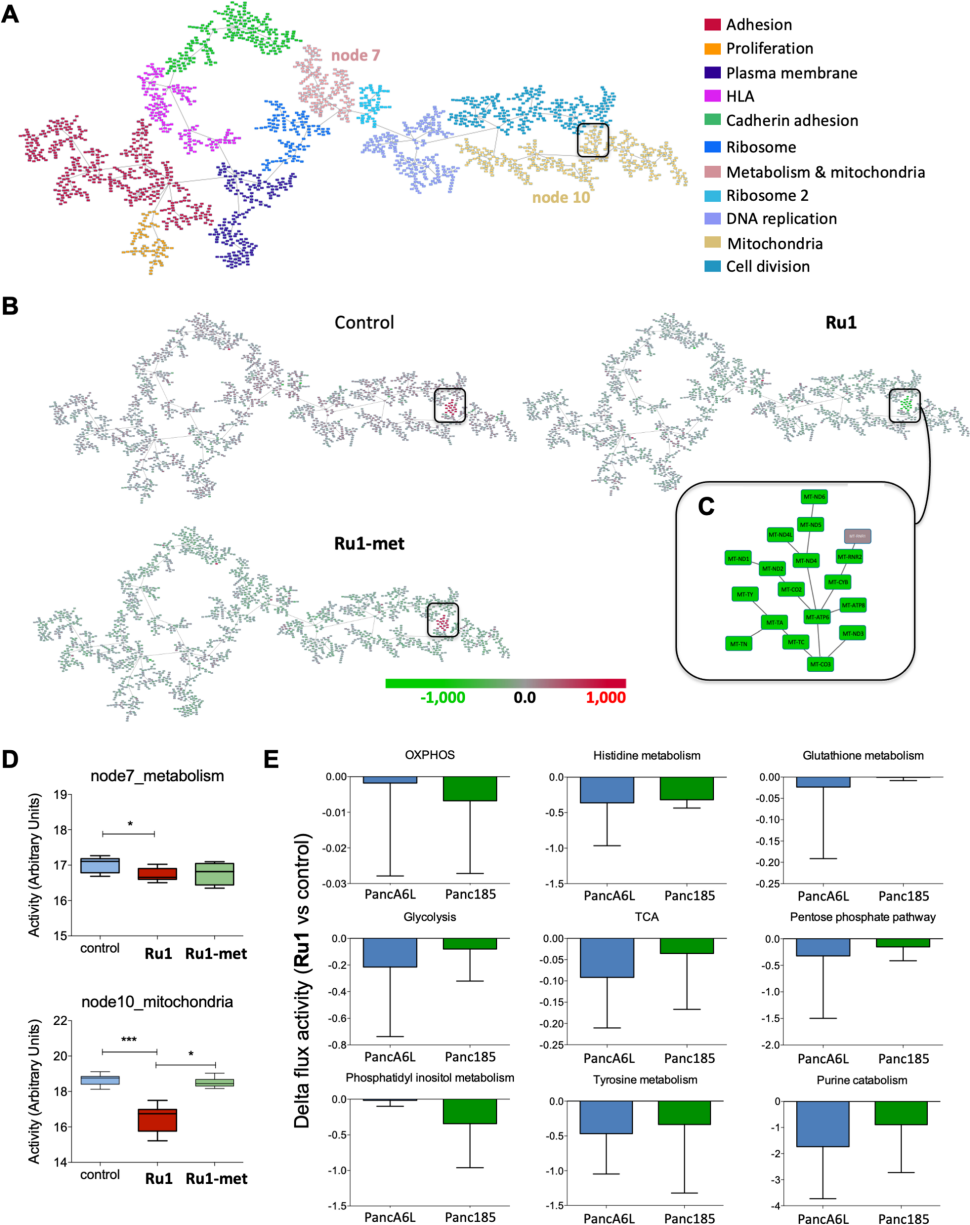
*Systems biology of pancreatic cancer and genes affected by Ru1.* **A** Resulting network derived from the probabilistic graphical model (PGM) analyses. Shown are the 11 functional color-coded nodes, determined based on the mean-centered expression for the 2,000 genes with the most significant variation. **B** Network Heatmap showing the genes with the greatest variability between *Ru1* and Control or *Ru1* and *Ru1-met*. Green=under expressed, Red= over expressed. **C** Genes located within the mitochondria node (Node 10) that are significantly decreased between *Ru1* and Control or *Ru1* and *Ru1-met*. **D** Functional activity of the nodes comparing Control, *Ru1* and *Ru1-met*. * *p* < 0.05, *** *p* < 0.001, as determined by one-way ANOVA with Tukey post-test. **E** Delta flux activities for *Ru1*-treated Panc185 or PancA6L cell compared to Control. In all cases, the indicated metabolic pathways decrease upon *Ru1* treatment

Alternatively, we performed a systems biology analysis of PDAC. The aforementioned RNAseq data, and mRNA expression data from our previously published studies [12], were combined (6 tumors, *n*=4-7 replicates per tumor) and used in a probabilistic graphical model analysis, with no other *a priori* information, as previously described [33]. The resulting network revealed functional structures, that is, mRNAs included in each branch of the tree had an overrepresentation in a biological function, resulting in 11 functional nodes identified, including nodes for metabolism and mitochondria (Fig. 7A). The mean-centered expression for the 2,000 genes included in the network was represented into the PGM network. Interestingly, this approximation highlighted the negative and specific effect of *Ru1* on gene expression in the mitochondrial node (based on 31 genes, Fig. 7B-C). We then established the level of activity of each functional node using the mean expression of all the mRNAs included in a given branch that belong to a common functional group. Next, we performed class comparison analyses to assess which functional nodes are differentially activated between control, *Ru1*- and *Ru1-met*-treated samples. Four nodes showed significant differences between groups. Of interest, Node 7 (Metabolism) and Node 10 (Mitochondria) showed significantly decreased activity in *Ru1*-treated cells (Fig. 7D), in line with our GSEA analysis.

Finally, in order to characterize the differences observed following treatment with *Ru1* at the level of metabolic pathways, a reconstruction of the human metabolism Recon3D [36] and the COBRA Toolbox [37] library, available for MATLAB, was used. The Recon3D contains information of 10,600 metabolic reactions, 5,835 metabolites and 5,938 Gene-Protein-Reaction rules (GPRs), which collects information on which genes are involved in each metabolic reaction. Once the flux activities were calculated, the delta flux value between *Ru1* versus control was calculated, confirming that *Ru1* clearly affects OXPHOS, but also touches other metabolic pathways (Fig. 7E).

### Ru1 affects CSC mitochondrial functions by inhibiting the transcription of mtDNA genes

**Fig. 8:**
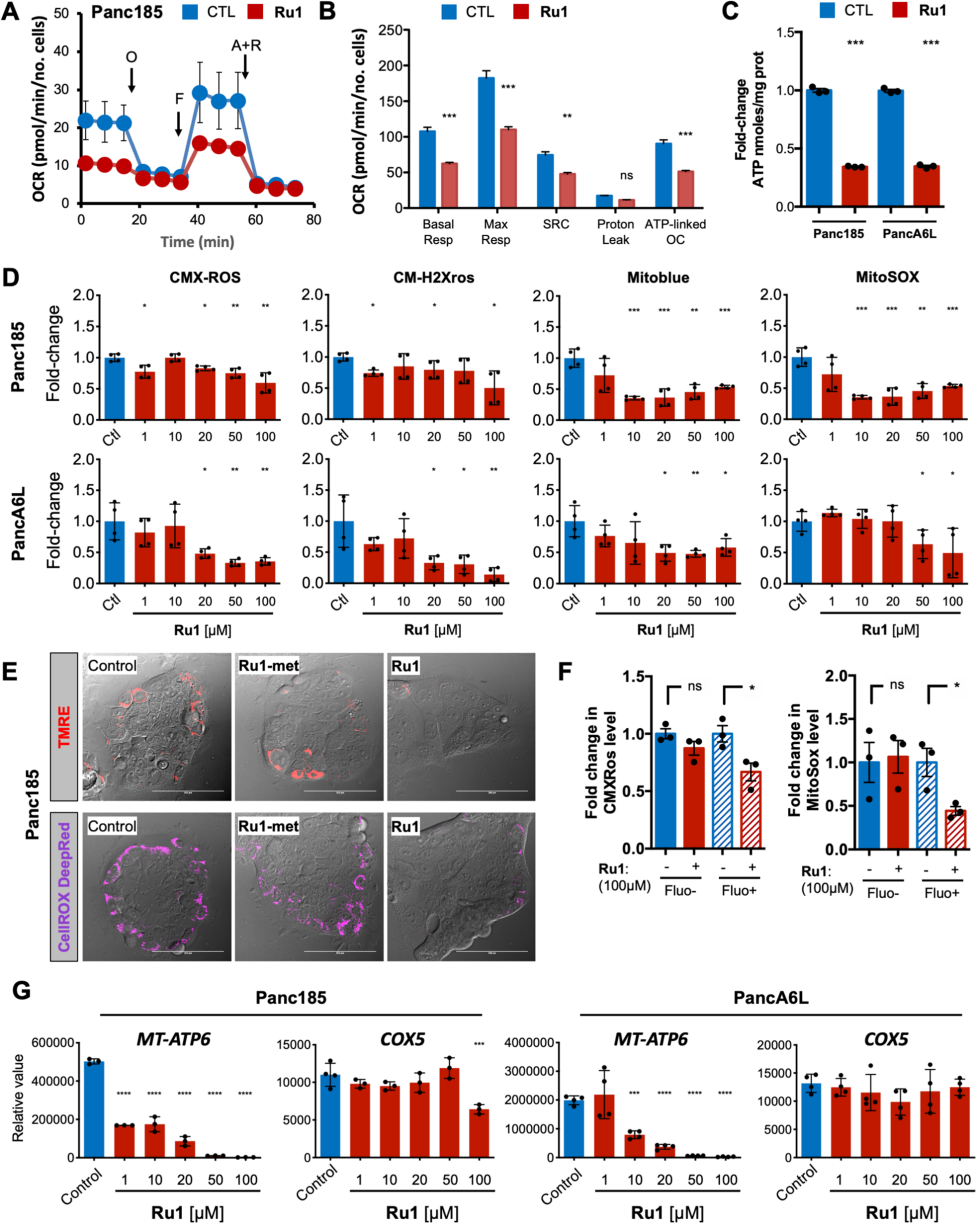
*Ru1 affects PaCSC oxygen consumption and mitochondrial functional properties.* **A** Representative plot showing mean ± sd of oxygen consumption rate (OCR) for untreated (CTL) and *Ru1*-treated Panc185 spheres (100µM, 24 h), normalized to total protein using a BCA kit, which were treated with distinct inhibitors of mitochondrial function: O (oligomycin), F (FCCP), A (antimycinA), and R (rotenone). Continuous OCR values (pmoles/min/no. cell) are shown. **B** Measured and calculated mean ± SD OCR parameters (Resp = Respiration; Max = Maximum; SRC = Spare Respiratory Capacity; OC = Oxygen consumption; *n*=3 biological replicates with 3 readings). ** *p* < 0.01, *** *p* < 0.001, ns = not significant, as determined by unpaired two-sided Student’s t-test. **C** Mean fold-change ± SD in ATP nmoles/mg protein in untreated (CTL) and *Ru1*-treated Panc185 and PancA6L (100µM, 24 h) cells compared to control, set as 1.0. *** *p* < 0.001, as determined by unpaired Student’s t test. **D** Mean fold-change ± SD in the mitochondria membrane potential probes CMX-ROS, CM-H2Xros and Mitoblue or the ROS probe MitoSOX as a function of increasing concentrations of *Ru1* in Panc185 or PancA6L cells (48h). * *p* < 0.05, ** *p* < 0.01, *** *p* < 0.001, as determined by one-way ANOVA with Dunnett post-test, compared to untreated (Ctl) set as 1.0. **E** Representative IF confocal images of TMRE (mitochondria membrane potential) or CellROX DeepRed (ROS) staining in untreated (Control), *Ru1* or *Ru1*-met-treated Panc185 cells (100µM, 24 h). **F** Mean fold-change ± SD in the mitochondria membrane potential probe CMX-ROS or the ROS probe MitoSOX in Autofluorescent-negative (Fluo-) or Fluo+ FACS-sorted cells pre-treated with *Ru1* (100µM, 48h). * *p* < 0.05, ns = not significant, as determined by unpaired Student’s t test. **G** Mean fold-change ± SD in the expression of the mtDNA-encoded gene *MTATP6* or the nuclear DNA encoded gene *COX5* as a function of increasing concentrations of *Ru1* in Panc185 or PancA6L cells (48h treatment). Values were normalized to ß-actin levels. *** *p* < 0.001, **** *p* < 0.0001, as determined by one-way ANOVA with Dunnett post-test, compared to untreated (Ctl)

Next, we validated the functional effect of *Ru1* on OXPHOS, and on other mitochondrial-related processes. To this end, we measured the oxygen consumption rate (OCR) of control- and *Ru1*-treated Panc185, PancA6L and Panc215 spheres, in the presence or absence of distinct inhibitors of mitochondrial function (Fig. 8A-B and S4A). Firstly, baseline OCR and maximal respiration (i.e., FCCP-stimulated respiration) were significantly lower in *Ru1***-**treated cells. Using these parameters to determine the spare respiratory capacity (SRC) (i.e. the difference between maximal respiration and basal OCR), we observed that SRC was significantly reduced in the presence of *Ru1,* indicating that treated cells are less able to overcome ATP demands under different types of mitochondrial stress [50]. In support of this claim, ATP-linked respiration was significantly lower (Fig. 8B and S4A), and ATP production was reduced in the cells treated with the active ruthenium complex (Fig. 8C). Moreover, we observed more lactate production when cells were treated with *Ru1* (Fig. S4B), indicating increased glycolysis, perhaps as a result of OXPHOS inhibition. Using probes for mitochondrial membrane potential (TMRE, CMX-ROS, CM-H2Xros and Mitoblue [25, 26]) or ROS production (CellROX DeepRed or MitoSOX), we observed that all indicators of mitochondrial function and ROS production decreased in *Ru1*-treated cells (Fig. 8D-E), when compared with controls and/or to cells treated with *Ru1-met* (Fig. 8E and S4C). These effects were accompanied by an accumulation of damaged and swollen mitochondria with less pronounced cristae (Fig. S4D-E). Likewise, when we separated PDAC cells using the CSC marker autofluorescence [46], the effects on mitochondrial function and ROS production were more pronounced in the CSC population (Fig. 8F). This reduction in mitochondrial function correlated perfectly with decreased transcription of the mtDNA-encoded gene *ATP6*, but not with the transcription of the gene *COX5* (except for Panc185 at 100µM) that also participates in the electron transport chain but is nuclear encoded, further supporting that *Ru1* works by inhibiting the transcription of mtDNA-encoded genes (Fig. 8G).

### Ru1 binds to the mtDNA D-loop and inhibits transcription

**Fig. 9:**
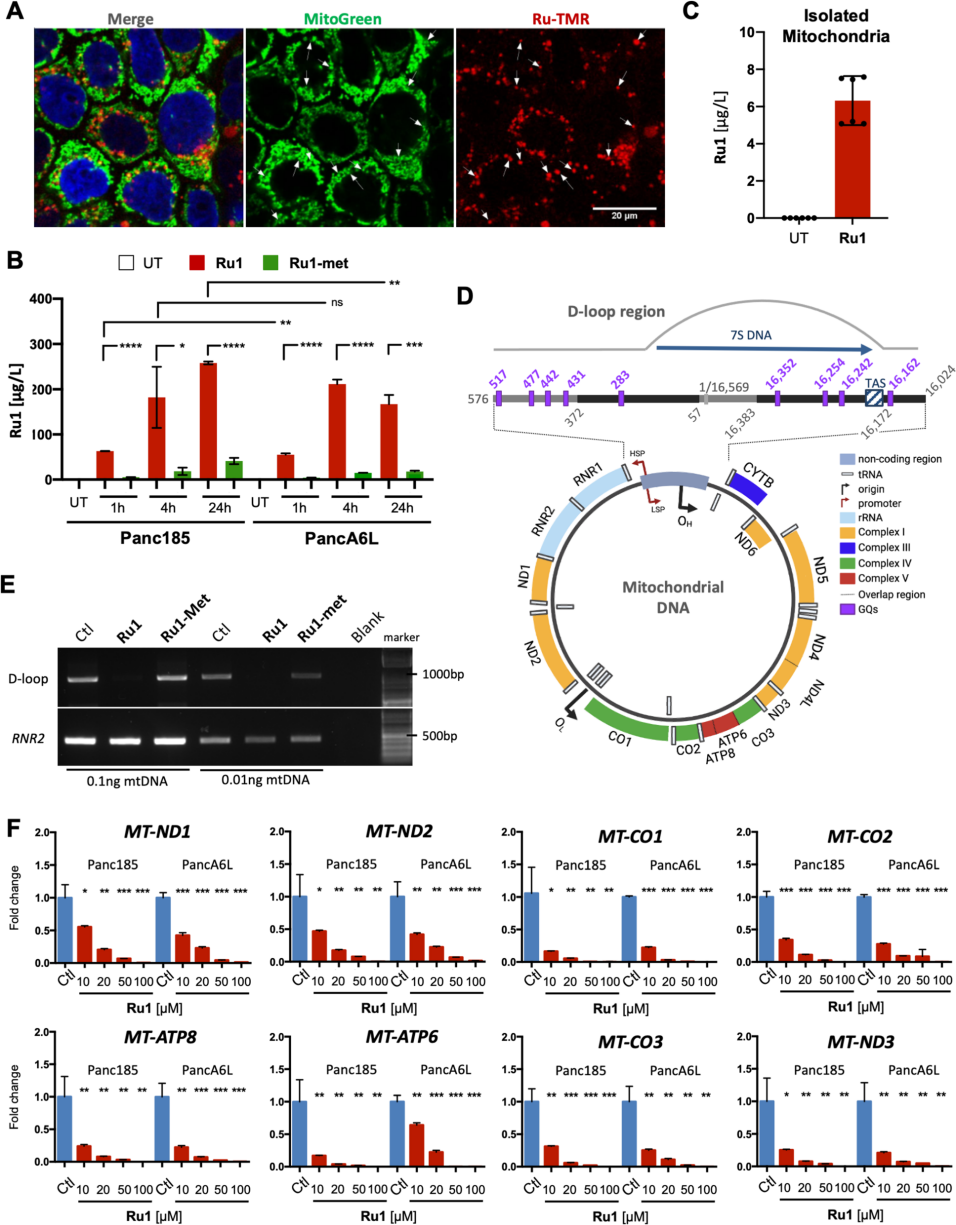
*Ru1 binds the mtDNA D-loop and inhibits transcription.* **A** Representative IF confocal images of MitoGreen (mitochondrial mass), *Ru1-TMR* (red) and DAPI (Blue) staining in *Ru1* (100µM)-treated Panc185 cells (24 h). White arrows indicate co-localization of *Ru1-TMR* and MitoGreen. Scale bar = 20 µm. **B** Amount of *Ru1* molecule (µg/L) in untreated (UT), *Ru1*-treated (red) or *Ru1-met*-treated (green) Panc185 and PancA6L, determined by ICP at the indicated times post-treatment with 100µM of compounds. **C** Amount of *Ru1* molecule (µg/L), determined by ICP, in gradient-purified mitochondria isolated from untreated (UT) and *Ru1*-treated Panc185 (100µM, 24 h). **D** Diagram of the mitochondrial genome, indicating the protein-coding genes (CI, yellow; CIII, blue, CIV, green; CV, red), and ribosomal RNA (rRNA)-coding genes (light blue). The D-loop region is magnified, and the predicted GQs and their positions are show in purple. TAS = termination associated sequence; HSP = heavy strand promoter; LSP = light strand promoter; O_H_ = origin of replication – heavy; O_L_ = origin of replication – light. Adapted from [52] and created in part with BioRender.com. **E** Agarose gel resolved D-loop or *RNR2* PCR products amplified from 0.1 or 0.01ng of mtDNA pre-incubated for 1 h with diluent (Ctl) or 10µM of *Ru1* or *Ru1-met*. **F** Mean fold-change ± SD in the expression of the indicated mtDNA-encoded genes as a function of increasing concentrations of *Ru1* in Panc185 or PancA6L cells (48 h treatment). Values were normalized to ß-actin levels. * *p* < 0.05, ** *p* < 0.01, *** *p* < 0.001, as determined by one-way ANOVA with Dunnett post-test, compared to untreated (Ctl) set as 1.0

The above data suggested that the effects of *Ru1* on PaCSC cells are in large part a consequence of a direct action at the level of their mitochondria. As already commented in the introduction, this type of metal polypyridine complex, by combining lipophilicity (of the ligands) and positive charge, tends to show a preferential ability to target mitochondria [20, 51]. Indeed, using the functional rhodamine-labeled analog *Ru-TMR* (Fig. 5B-C), confocal fluorescence microscopy confirmed that *Ru-TMR* is efficiently internalized into Panc185 cells, with a localization to mitochondria (Fig. 9A). Moreover, using TMR-unlabeled Ru compounds, ICP-MS analysis of cell lysates from Panc185 cells incubated with *Ru1* or *Ru1-met* confirmed cellular internalization, with *Ru1* showing superior internalization (Fig. 9B), compared to *Ru1-met*. Moreover, *Ru1* efficiently accumulated in mitochondria (Fig. 9C), and specifically interacted with the mtDNA (Fig. S4F), suggestive of a strong coordinating interaction with the mtDNA.

Considering our previous *in vitro* studies [15], the association of *Ru1* to the mtDNA might be due to presence of unpaired, solvent accessible guanines, probably in adjacent positions to secondary GQ-type structures. Using the G4 hunter software to scan the mtDNA [53], we found 109 sites (Fig. S5), of which 82% were present in the protein coding regions of both the L-strand and H-strands, with a greater number of sequences identified in the H-strand. Interestingly, the 18% remaining sequences identified by G4 hunter were located in the non-coding regions (<5% of the mtDNA), with 45% concentrating in the D-loop region (Fig. 9D, S5 and S6A), an area of the mtDNA that contains the main regulatory sites for transcription initiation [54]. Therefore, it is reasonable to hypothesize that the effect of *Ru1* might derive from a direct reaction with accessible guanines present in the D-loop region.

To gain further insights, we incubated 0.1 and 0.01 ng of purified mtDNA with 10µM *Ru1* for 1 h and performed a PCR to amplify specific regions of 1) the D-loop: a 1146 bp region (mtDNA positions 16,067 to 644 bp), containing 9 putative GQs, and 2) a region within the RNR2 gene that does not contain potential GQs (2091 – 2640 bp). Remarkably, we found that *Ru1* halted Taq polymerase-mediated amplification of the D-loop region but not of the RNR2 region (Fig. 9E).

**Fig. 10:**
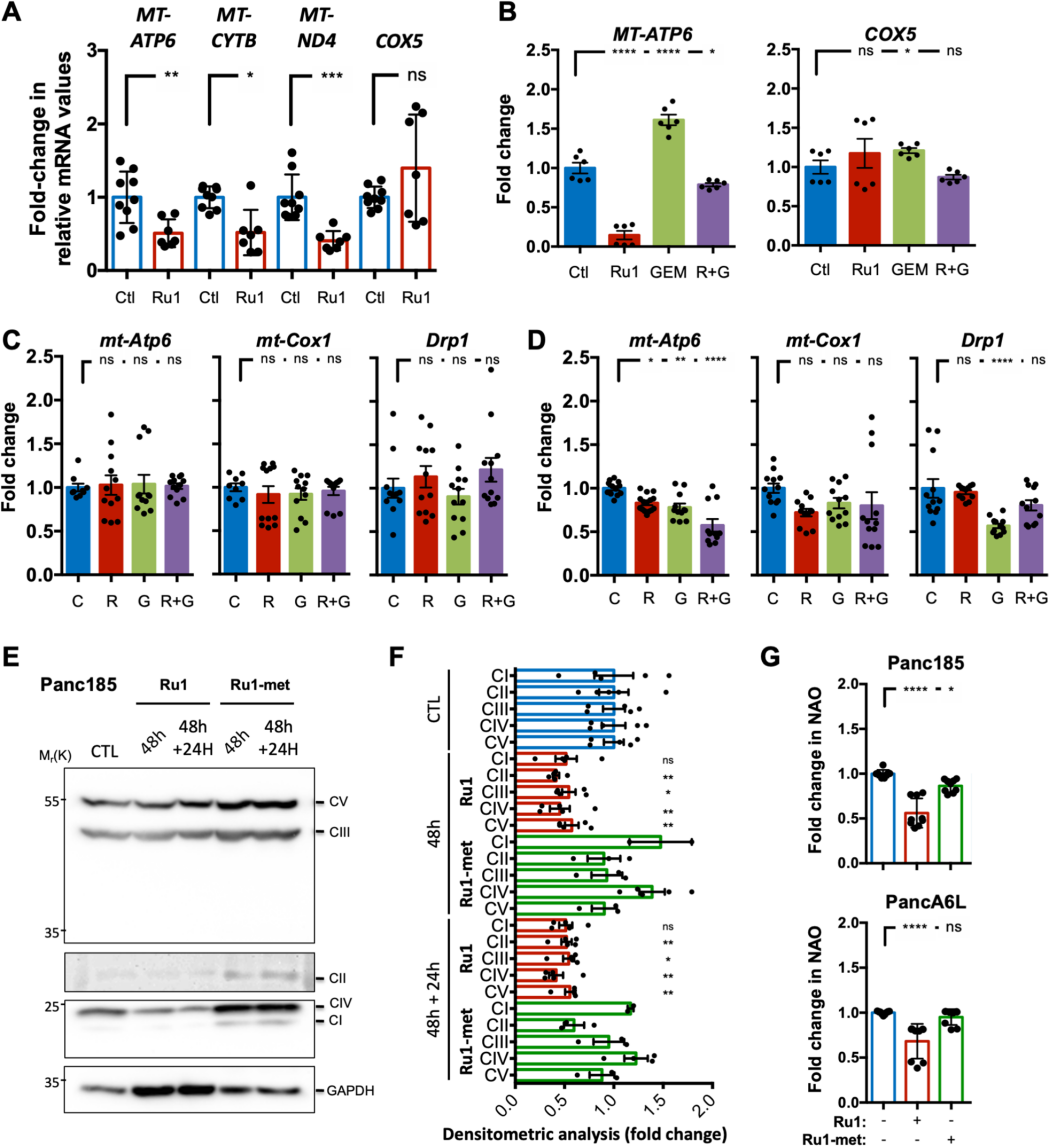
*Ru1 inhibits mtDNA transcription, OXPHOS protein complex translation and mitochondrial mass.* **A** Mean fold-change ± SD in the expression of the mtDNA-encoded gene *MTATP6*, *MTCYTB* or *MTND4* and the nuclear DNA encoded gene *COX5* in Panc354 tumors extracted on d19 from mice treated with Ctl or *Ru1* (1.4mg/kg; daily, r.o.). Values were normalized to ß-actin levels. * *p* < 0.05, ** *p* < 0.01, *** *p*<0.001, ns = not significant, as determined by unpaired two-sided Student’s t-test. **B** Mean fold-change ± SEM in the expression of the mtDNA-encoded gene *MTATP6* or the nuclear DNA encoded gene *COX5* in Panc265 orthotopic tumors extracted from mice 3 weeks post treatment initiation with Control (Ctl) *Ru1*, gemcitabine (50mg/kg; twice per week) GEM or a combination of both (R+G). Values were normalized to ß-actin levels. * *p* < 0.05, **** *p* < 0.0001, ns = not significant, as determined by one-way ANOVA with Dunnett post-test, compared to untreated (Ctl). **C-D** Mean fold-change ± SEM in the expression of murine mtDNA-encoded genes *mt-Atp6* and *mt-Cox1* or the nuclear DNA encoded gene *Drp1* in the heart (**C**) or liver (**D**) from mice harboring Panc265 orthotopic tumors, 3 weeks post treatment initiation with Control (C), *Ru1* (R), gemcitabine (50mg/kg; twice per week) (G), or a combination of both (R+G). Values were normalized to *Hprt* levels. * *p* < 0.05, ** *p* < 0.01, **** *p* < 0.0001, ns = not significant, as determined by one-way ANOVA with Dunnett post-test, compared to untreated (Ctl). **E** WB analysis of mitochondria OXPHOS complex proteins using the Mitoprofile Total OXPHOS antibody cocktail in addition to GAPDH (loading control). Shown are bands corresponding to Complex (C)V, CIII, CII, CIV, and CI from Panc185 cells that were either untreated (CTL) or treated with *Ru1* (100µM) or *Ru1-met* (100µM) for 48 h and 48 h + 24 h after removing treatment. **F** Mean fold-change ± SEM of mitochondrial complex bands determined in (**E**) by densitometric analysis and normalized to GAPDH. (*n*=4 pooled WBs, * *p* < 0.05, ** *p* < 0.01, ns = not significant, as determined by one-way ANOVA with Dunnett post-test, compared to untreated (CTL). **G** Mean fold-change ± SD in the mitochondria mass probe NAO in untreated (-), *Ru1* (100µM) or *Ru1-met* (100µM)-treated Panc185 and PancA6L cells (48 h). * *p* < 0.05, **** *p* < 0.0001, ns = not significant, as determined by one-way ANOVA with Dunnett post-test, compared to untreated (Ctl)

This interesting result suggests a direct and stable modification of the DNA sequence corresponding to the D-loop, which impairs polymerase amplification, and may involve a coordination to specific solvent accessible guanines. Indeed, when using *Ru1-met*, which lacks the coordination position required for interaction with guanines, the inhibition was not observed (Fig. 9E). The sum of these results further reinforced a mechanism of action entailing a direct metalating coordination of *Ru1* to accessible guanines in the D-loop region of the mtDNA, a physical interaction that explains the observed inhibition of key mitochondrial genes, not only *MT-ATP6* (Fig. 8G), but all 13 mtDNA protein-coding genes in Panc185 and PancA6L cells (Fig. 9F and S6B). Remarkably, there was no appreciable effect on the transcription of nuclear-encoded OXPHOS-related genes (i.e., *COX5*, *NDUFA* and *UQCRC2*) (Fig. S6B), confirming a selective action on the mtDNA. This effect on mtDNA was also validated in samples collected from the *in vivo* intervention studies (Fig. 3). In mice treated with *Ru1* there was a significant decrease in the transcription of mtDNA-encoded human genes (Fig. 10A-B), and the effect was tumor specific as *Ru1* did not reduce murine mtDNA or nuclear transcript levels (i.e., *mt-Atp6, mt-Cox1* and *Drp1*) in the heart or liver of treated mice (Fig. 10C-D), confirming tumor selectivity. Moreover, OXPHOS complex proteins, as determined by WB analysis, were reduced approximately 50% following treatment with *Ru1* (Fig. 10E-F) and a significant reduction in mitochondrial mass, using the membrane-potential-independent dye NAO, was also observed (Fig. 10G). Altogether, these data strongly support the conclusion that *Ru1* acts at the level of the mtDNA, inhibiting mtDNA transcription, which leads to reduced OXPHOS, OXPHOS complex proteins, and mitochondrial mass *in vitro* and/or *in vivo*.

## Discussion

Cis-platinum and derivatives are reactive metal complexes that have shown impressive utility as anti-cancer chemotherapeutic agents by inducing cancer cell apoptosis. However, these compounds present a promiscuous reactivity, and hence elicit many secondary toxic and resistance effects [55]. Attempts have been made to develop related anti-cancer metal complexes with better selectivity than platinum derivatives, and in this context, ruthenium has been especially attractive owing to the ligand tuning possibilities and accessible coordination geometries. In fact, two ruthenium complexes (NAMI-A and NKP1339) have even entered clinical trials [56], although apparently, they work by targeting proteins and/or altering the cellular redox state rather than by interacting with DNA. However, as with most metallodrugs, these ruthenium complexes are also quite promiscuous in terms of reactivity, which makes it difficult to control their biological fate and targeting profile [57, 58].

Therefore, a major challenge in the field has been the discovery of metallo-derivatives with kinetically controlled reactivity and increased selectivity with regard to their biological targets. In this context, we recently found that the ruthenium complex *Ru1* is capable of metalating solvent exposed guanine residues, such as those present in adjacent positions of GQs, with high selectivity and low toxicity [15]. This curious combination of controlled reactivity with DNA and lack of cytotoxicity, prompted us to explore its potential biological applications. We have now discovered that *Ru1* exhibits a potent inhibitor effect on PaCSCs, by targeting genes involved in OXPHOS. More importantly, the compound exerts impressive anticancer activity in vivo. Preclinical evaluation of *Ru1* in 6 different subcutaneous PDX models of PDAC, including an orthotopic PDAC tumor model, as well as CRC and OS PDXs, showed potent cytostatic activity, inhibiting tumor proliferation as early as 1-2 days post treatment initiation. This effect is comparable to what others have accomplished using toxic combination therapies (e.g., inhibitors that target upstream (SHP2 or SOS1) and downstream (MEK) mediators of KRAS signaling [59, 60]), but without the toxic or resistance-associated side-effects. Moreover, while an additive effect was observed for some tumors when *Ru1* was combined with gemcitabine, reduced tumor re-growth was observed for all PDAC tumors treated with the combination approach compared to gemcitabine alone, which we attribute to a reduction in the non-CSC population as well as the CSC population, the latter being the drivers of disease relapse.

Regarding the mechanism of action, control experiments with *Ru1-met* or *Ru1-py* (analogs of *Ru1* that lack a kinetically labile coordination position) revealed that these compounds are inert under the same conditions, suggesting that the activity of *Ru1* is mediated by displacement of the labile aquo ligand by some nucleophilic component of a biological molecule, most probably nucleic acid guanines. Indeed, detailed mechanistic experiments with PaCSCs revealed that *Ru1* can reach the mitochondria and interact with their mtDNA. We were able to map this interaction to the D-loop region, an area of the mtDNA that contains the main regulatory sites for transcription initiation [54]. Consequently, RNAseq analysis of PancA6L CSCs treated with *Ru1* showed modulation of only mtDNA encoded transcripts, suggesting that the functional effect of *Ru1* is indeed mitochondriotropic. Nonetheless, we cannot completely rule out that *Ru1* could interact with other regions of the mtDNA and/or nuclear DNA, although confocal microscopy analysis of PaCSCs treated with Ru-TMR did not show signal in the nuclei (Fig. 9A). Along these lines, Panc185 cells showed modulation of more genes compared to PancA6L in our RNAseq analyses, including nuclear genes that encode OXPHOS components (e.g., *COX5*), but only at concentrations of 100µM (Fig. 8G). These differences between Panc185 and PancA6L may be due to differences in the amount of *Ru1* that enters each cell line, with Panc185 up taking more *Ru1* over time (Fig. 9B). Thus, while we cannot exclude other mechanisms of action contributing to the biological effects observed in this study, it is clear that *Ru1* reduces the mRNA of all 13 mtDNA protein-encoding genes, which provokes a decrease in oxygen consumption, mitochondrial membrane potential, and ATP production, as well as a decrease on the members of the OXPHOS complex, all of which are necessary for PaCSCs, which depend on mitochondrial respiration to meet their energy requirements and are therefore more susceptible to mitochondriotropics compared to non-CSCs [12, 61]. Examples of other inorganic complexes that work as mitochondriotropics have been described [21, 62–64]; however, their anti-cancer activity is associated to mitochondrial-induced apoptosis, very different than that of *Ru1*, which at the concentrations used in this study do not induce apoptosis (Fig. 1H) or increase ROS (Fig. 8D). Organic compounds, such as the benzene-1,4-disulfonamide compound 23 (DX3–213B), have shown promising results at the level of tumor growth inhibition in a PDAC syngeneic in vivo model, by disrupting ATP generation; however, its mechanism of action has not been elucidated, but most likely it is not mediated by inhibiting CSCs [65].

## Conclusions

*Ru1* can certainly be considered a mitochondriotropic, functioning via a novel mechanism of action consisting in the inhibition of mtDNA gene transcription. Importantly, and as mentioned above, it neither increases ROS levels nor induces apoptosis or toxicity in any of the assays performed in vitro or in vivo. Thus, the ruthenium complex *Ru1* is not only a potent non-toxic mitochondriotropic, but it is an excellent anti-CSC agent as it targets a necessary CSC bioenergetic pathway, representing a promising new lead for the treatment of PDAC and other cancers driven by OXPHOS-dependent CSCs. To the best of our knowledge, this type of mechanism is unprecedented, and therefore this inorganic compound represents not only an exciting new anti-cancer agent, but also a relevant tool from a cell biology perspective to dissect the role of OXPHOS in CSCs. The preliminary experiments indicating that the compound is effective *in vivo* are extremely exciting, and of high preclinical value.

### Supplementary Information

**Additional file 1:** **Figure S1.** NMR studies of the aquation of [Ru(terpy)(bpy)Cl]Cl complex (Ru0) using deuterium oxide. (A) ^1^H-NMR of [Ru(terpy)(bpy)Cl]Cl complex (*Ru0*) in water at t= 0. (B) ^1^H-NMR of [Ru(terpy)(bpy) H2O]^+2^Cl^-2^ complex (*Ru1*) in water. (C) ^1^H-NMR of [Ru(terpy)(bpy)Cl]Cl complex (*Ru0*) in water at different times (starting concentration of *Ru0* = 2mM). (D) ^1^H-NMR of [Ru(terpy)(bpy)Cl]Cl complex (*Ru0*) after dissolving in water (t = 0 min) and after irradiation with visible light for 60-120 min (starting concentration of *Ru0*= 2mM). **Figure S2.** Analysis of *Ru1* toxicity in vivo. (A-C) Average values ± SEM of indicated hematocrit parameters determined from blood of mice extracted 2h (A), 4h (B), or 8h (C) post treatment with diluent control (Ctl) or *Ru1*(1.4mg/kg, r.o). No significant differences were found, as determined by unpaired two-sided Student’s t-test. (D) Picomoles of *Ru1* per mg of tumor, determined by ICP-MS, from tumors extracted at indicated time points post treatment initiation. Dashed line indicates the background of the assay. **Figure S3.** *Ru1* negatively regulates MT-encoded genes. (A) Table summarizing the 14 mtDNA-encoded genes modulated in PancA6L spheres treated with *Ru1*(100μM, 24 h) compared to untreated Controls. Shown are the gene name, description, Log2 fold change, *p* value and *p* adjusted (adj). (B) Mean ± SD of normalized Fragments Per Kilobase Million (FPKM) values for the indicated target genes in Ctl-, *Ru1*and *Ru1-met*-treated Panc185 or PancA6L spheres. (ns=not significant, as determined by one-way ANOVA with Dunnett post-test, compared to Control). **Figure S4.** *Ru1* affects PaCSC oxygen consumption and mitochondrial properties and morphology. (A) Measured and calculated mean ± SD oxygen consumption rate (OCR) parameters (Resp = Respiration; Max = Maximum; SRC = Spare Respiratory Capacity; OC =Oxygen consumption) in Ctl- and *Ru1*-treated PancA6L or Panc215 spheres (n = 3 biological replicates with 3 readings). * *p* < 0.05, ** *p* < 0.01, *** *p* < 0.001, **** *p* < 0.0001, ns = not significant, as determined by unpaired two-sided Student’s t-test. (B) Mean fold-change ± SD in lactate (mM/total protein) in untreated (-), *Ru1*(100μM) or *Ru1-met* (100μM)-treated Panc185 and PancA6L cells compared to control, set as 1.0. *** *p* < 0.001, ns = not significant, as determined by one-way ANOVA with Dunnett post-test, compared to Control. (C) Representative IF confocal images of TMRE (mitochondria membrane potential) or CellROX DeepRed (ROS) staining in untreated (Control), *Ru1*(100μM) or *Ru1-met*(100 μM)-treated PancA6L cells (24 h). (D) Representative fluorescence confocal images of MitoGreen (mitochondrial mass) and DAPI (Blue) staining in *Ru1*(100μM)- treated PancA6L cells (24 h). Scale bar = 10 μM. (E) Representative transmission electron micrographs of Control (untreated) or *Ru1*(100μM)-treated Panc185 cells (24 h). Mitochondria are better defined in the Control compared to *Ru1*-treated samples. (F) Amount of *Ru1* molecules per 1000bp of DNA, determined by ICP, in mtDNA isolated from gradient-purified mitochondria from untreated (CTL) or *Ru1*-treated (100μM, 2 h) Panc185 cells. **Figure S5.** GQ in the mtDNA determined with G4 hunter. **Figure S6.** *Ru1* inhibits mtDNA transcription. (A) Sequence of 9 predicted GQs, their positions and G4Hunter score indicating G4-prone structures. Isolated guanines (G) are shown in red, and cysteines (C) in blue. (B) Mean fold-change ± SD in the relative mRNA expression of the indicated mtDNA- and nuclear-encoded genes as a function of increasing concentrations of *Ru1* in Panc185 or PancA6L cells (48h treatment). Values were normalized to ß-actin levels. * *p* < 0.05, ** *p* < 0.01, *** *p* < 0.001, as determined by one-way ANOVA with Dunnett post-test, compared to untreated (Ctl). **Table S1.** Antibodies. **Table S2.** RTqPCR primer sequences. **Table S3.** PCR primer sequences.

## Data Availability

RNAseq data generated in this study have been deposited in the SRA database (https://www.ncbi.nlm.nih.gov/sra/PRJNA832709) under accession number PRJNA832709. Unique identifiers for publicly available datasets are indicated. Reasonable requests for source data, resources and reagents should be directed to and will be fulfilled by the corresponding author.

## Abbreviations

CSCs: Cancer stem cells
PDAC: Pancreatic ductal adenocarcinoma
OXPHOS: Oxidative phosphorylation
mtDNA: Mitochondrial DNA
PaCSCs: Pancreatic CSCs
PDXs: Patient-derived xenografts
Ru1: [Ru(terpy)(bpy)(H_2_O)]Cl_2_
Ru0: [Ru(terpy)(bpy)Cl]Cl
CNIO: Spanish National Cancer Centre
LDA: Limiting Dilution Analysis
GTC: Guanidine thiocyanate
EE: Energy Expenditure
5FU: Fluorouracil
SRC: Spare respiratory capacity
GQ: g-quadruplex
