# Supplementary material for: Targeting cancer stem cell OXPHOS with tailored ruthenium complexes as a new anti-cancer strategy
Source: J Exp Clin Cancer Res. 2024 Jan 27;43:33. doi: 10.1186/s13046-023-02931-7 (PMC10821268; doi:10.1186/s13046-023-02931-7)
Supplement: Supplementary file 1 — Additional file 1: Figure S1. NMR studies of the aquation of [Ru(terpy)(bpy)Cl]Cl complex (Ru0) using deuterium oxide. (A) 1H-NMR of [Ru(terpy)(bpy)Cl]Cl complex (Ru0) in water at t= 0. (B) 1H-NMR of [Ru(terpy)(bpy) H2O]+2Cl-2 complex (Ru1) in water. (C) 1H-NMR of [Ru(terpy)(bpy)Cl]Cl complex (Ru0) in water at different times (starting concentration of Ru0 = 2mM). (D) 1H-NMR of [Ru(terpy)(bpy)Cl]Cl complex (Ru0) after dissolving in water (t = 0 min) and after irradiation with visible light for 60-120 min (starting concentration of Ru0= 2mM). Figure S2. Analysis of Ru1 toxicity in vivo. (A-C) Average values ± SEM of indicated hematocrit parameters determined from blood of mice extracted 2h (A), 4h (B), or 8h (C) post treatment with diluent control (Ctl) or Ru1(1.4mg/kg, r.o). No significant differences were found, as determined by unpaired two-sided Student’s t-test. (D) Picomoles of Ru1 per mg of tumor, determined by ICP-MS, from tumors extracted at indicated time points post treatment initiation. Dashed line indicates the background of the assay. Figure S3. Ru1 negatively regulates MT-encoded genes. (A) Table summarizing the 14 mtDNA-encoded genes modulated in PancA6L spheres treated with Ru1(100μM, 24 h) compared to untreated Controls. Shown are the gene name, description, Log2 fold change, p value and p adjusted (adj). (B) Mean ± SD of normalized Fragments Per Kilobase Million (FPKM) values for the indicated target genes in Ctl-, Ru1and Ru1-met-treated Panc185 or PancA6L spheres. (ns=not significant, as determined by one-way ANOVA with Dunnett post-test, compared to Control). Figure S4. Ru1 affects PaCSC oxygen consumption and mitochondrial properties and morphology. (A) Measured and calculated mean ± SD oxygen consumption rate (OCR) parameters (Resp = Respiration; Max = Maximum; SRC = Spare Respiratory Capacity; OC =Oxygen consumption) in Ctl- and Ru1-treated PancA6L or Panc215 spheres (n = 3 biological replicates with 3 readings). * p < 0.05, ** p < 0.01, *** p [file 13046_2023_2931_MOESM1_ESM.pdf]

Targeting cancer stem cell OXPHOS with tailored ruthenium complexes as a new anti-cancer strategy

Sonia Alcalá[1,2], Lara Villarino[3], Laura Ruiz-Cañas[1,2], José R. Couceiro[3], Miguel Martínez-Calvo[3], Adrián Palencia-Campos[1,2], Diego Navarro[1,2], Pablo Cabezas-Sainz[5], Iker Rodriguez-Arabaolaza[1,4], Alfonso Cordero-Barreal[1,2], Lucia Trilla-Fuertes[6,7], Juan A. Rubiolo[5], Sandra Batres-Ramos[1,2], Mireia Vallespinos[1,2], Cristina González-Páramos[1,8,9], Jéssica Rodríguez[3], Angelo Gámez-Pozo[6,7], Juan Ángel Fresno Vara[6,10], Sara Fra Fernández[11], Amparo Benito Berlinches[2,12], Nicolás Moreno Mata[11], Ana María Torres Redondo[13], Alfredo Carrato[2,10], Patrick C. Hermann[14], Laura Sánchez[5], Susana Torrente[15], Miguel Ángel Fernández-Moreno[1,8,9], José L. Mascareñas[3,*] and Bruno Sainz, Jr.[1,2,10,*]

Supplementary Material

Supplementary Figures S1-S6

Supplementary Tables S1-S3

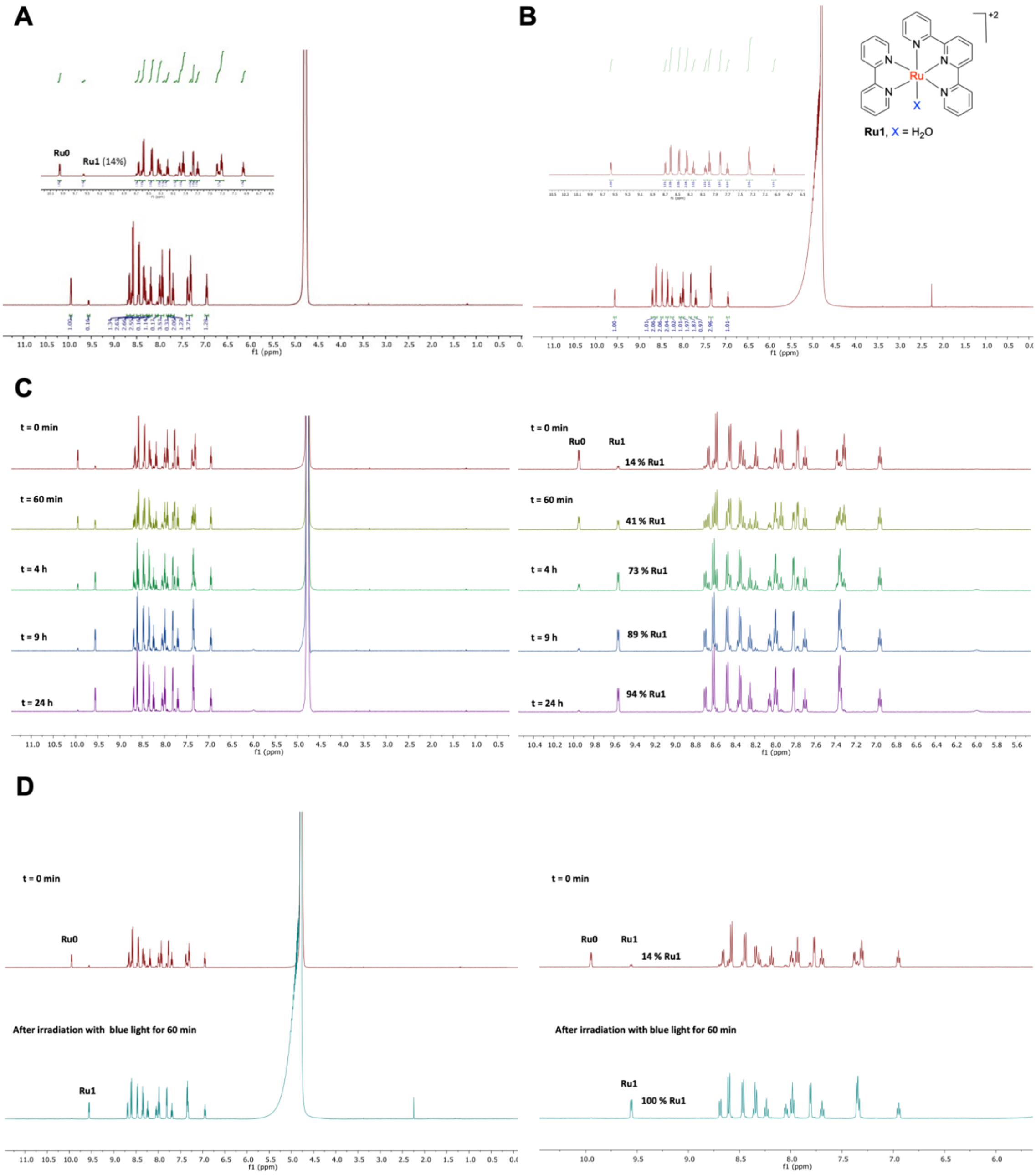

Fig. S1 *NMR studies of the aquation of [Ru(terpy)(bpy)Cl]Cl complex (Ru0) using deuterium oxide.* **A** ^{1}H-NMR of [Ru(terpy)(bpy)Cl]Cl complex (Ru0) in water at t= 0. **B** ^{1}H-NMR of [Ru(terpy)(bpy) $H_2O]^{+2}Cl^{-2}$ complex (*Ru1*) in water. **C** ^{1}H-NMR of [Ru(terpy)(bpy)Cl]Cl complex (Ru0) in water at different times (starting concentration of Ru0 = 2mM). **D** ^{1}H-NMR of [Ru(terpy)(bpy)Cl]Cl complex (*Ru0*) after dissolving in water (t = 0 min) and after irradiation with visible light for 60-120 min (starting concentration of *Ru0* = 2mM).

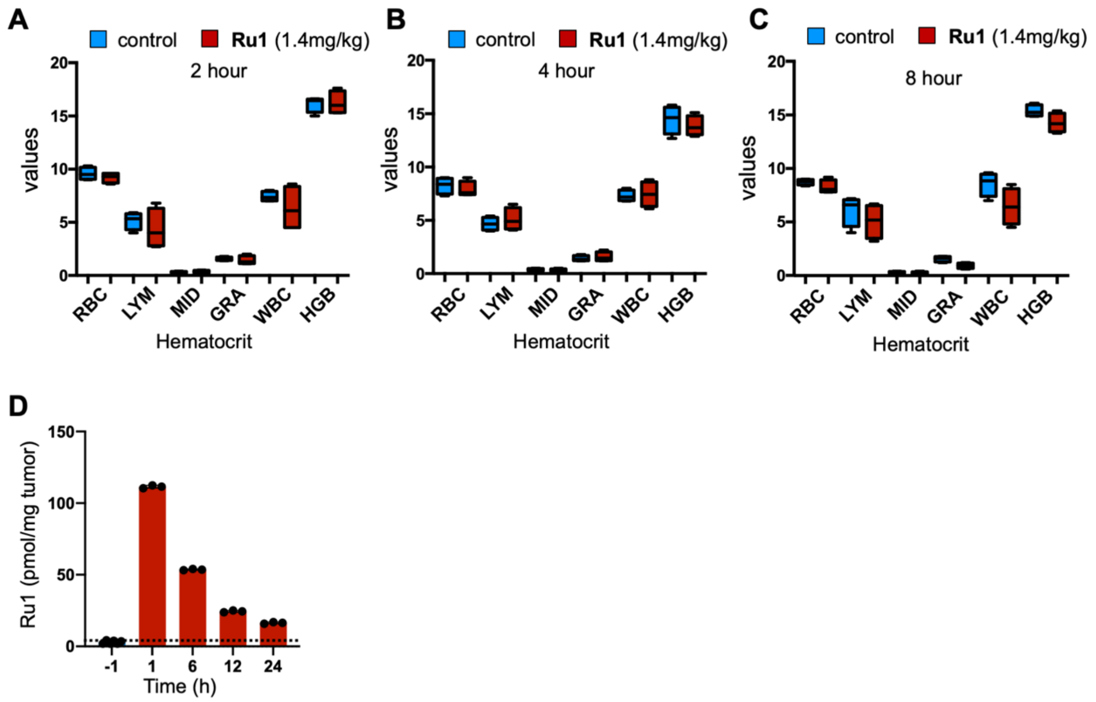

Fig. S2 *Analysis of Ru1 toxicity in vivo.* **A-C** Average values ± SEM of indicated hematocrit parameters determined from blood of mice extracted 2h (**A**), 4h (**B**), or 8h (**C**) post treatment with diluent control (Ctl) or *Ru1* (1.4mg/kg, r.o). No significant differences were found, as determined by unpaired two-sided Student's t-test. **D** Picomoles of *Ru1* per mg of tumor, determined by analyzing ruthenium with ICP-MS, from tumors extracted at indicated time points post treatment initiation. Dashed line indicates the background of the assay.

A

Gene	Description	Log2 Fold Change	p value	p adj
MT-ND5	MT **Encoded** NADH:Ubiquinone Oxidoreductase Core Subunit 5	**-4.583453047**	2.727E-21	2.507E-17
MT-ND4L	MT **Encoded** NADH:Ubiquinone Oxidoreductase Core Subunit 4L	**-4.139355065**	1.339E-19	6.842E-16
MT-ND6	MT **Encoded** NADH:Ubiquinone Oxidoreductase Core Subunit 6	**-4.533419675**	1.488E-19	6.842E-16
MT-ND4	MT **Encoded** NADH:Ubiquinone Oxidoreductase Core Subunit 4	**-4.79740817**	3.726E-19	1.370E-15
MT-CYB	MT **Encoded** Cytochrome B	**-4.546259595**	4.588E-19	1.406E-15
MT-ATP8	MT ATP Synthase Membrane Subunit 8	**-3.980573335**	1.279E-15	3.359E-12
MT-CO1	MT **Encoded** Cytochrome C Oxidase I	**-4.436182642**	2.545E-13	5.200E-10
MT-CO3	MT **Encoded** Cytochrome C Oxidase 3	**-4.162005073**	7.644E-13	1.405E-09
MT-ATP6	MT ATP Synthase Membrane Subunit 6	**-4.127331673**	1.441E-12	2.408E-09
MT-CO2	MT **Encoded** Cytochrome C Oxidase 2	**-4.009943774**	8.000E-09	1.131E-05
MT-ND3	MT **Encoded** NADH:Ubiquinone Oxidoreductase Core Subunit 3	**-4.683257976**	4.332E-07	5.310E-04
MT-TF	MT **Encoded** TRNA-Phe (UUU/C)	**3.177659894**	3.776E-06	4.340E-03
MT-TS1	MT **Encoded** TRNA-Ser (UCN) 1	**-4.245403579**	5.998E-06	6.487E-03
MT-TC	MT **Encoded** TRNA-Cys (UGU/C)	**-4.141347134**	1.300E-05	0.0125823

B

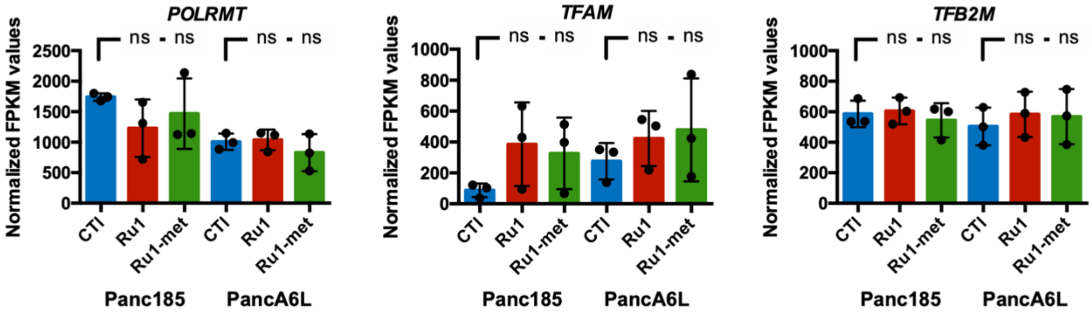

Fig. S3 *Ru1 negatively regulates MT-encoded genes.* **A** Table summarizing the 14 mtDNA-encoded genes modulated in PancA6L spheres treated with *Ru1* (100µM, 24 h) compared to untreated Controls. Shown are the gene name, description, Log2 fold change, *p* value and *p* adjusted (adj). **B** Mean ± SD of normalized Fragments Per Kilobase Million (FPKM) values for the indicated target genes in Ctl-, *Ru1* and *Ru1-met*-treated Panc185 or PancA6L spheres. (ns= not significant, as determined by one-way ANOVA with Dunnett post-test, compared to Control).

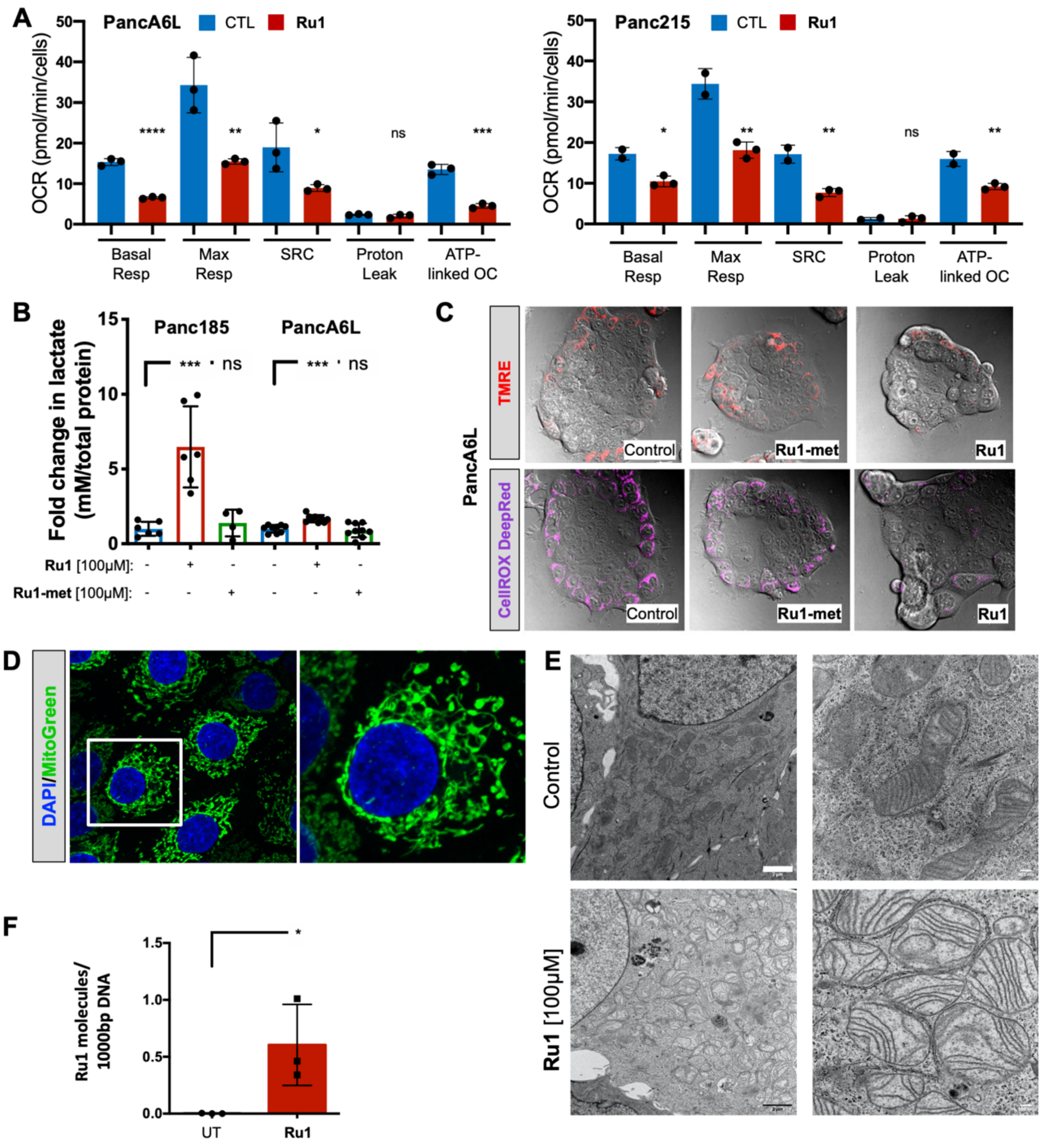

Fig. S4 *Ru1 affects PaCSC oxygen consumption and mitochondrial properties and morphology.* **A** Measured and calculated mean ± SD oxygen consumption rate (OCR) parameters (Resp = Respiration; Max = Maximum; SRC = Spare Respiratory Capacity; OC = Oxygen consumption) in Ctl- and *Ru1*-treated PancA6L or Panc215 spheres (n = 3 biological replicates with 3 readings). * $p < 0.05$, ** $p < 0.01$, *** $p < 0.001$, **** $p < 0.0001$, ns = not significant, as determined by unpaired two-sided Student's t-test. **B** Mean fold-change ± SD in lactate (mM/total protein) in untreated (-), *Ru1* (100μM) or *Ru1-met* (100μM)-treated Panc185 and PancA6L cells compared to control, set as 1.0. *** $p < 0.001$, ns = not significant, as determined by one-way ANOVA with Dunnett post-test, compared to Control. **C** Representative IF confocal images of TMRE (mitochondria membrane potential) or CellROX DeepRed (ROS) staining in untreated (Control), *Ru1* (100μM) or *Ru1-met* (100μM)-treated PancA6L cells (24 h). **D** Representative fluorescence confocal images of MitoGreen (mitochondrial mass) and DAPI (Blue) staining in *Ru1* (100μM)-treated PancA6L cells (24 h). Scale bar = 10 μM. **E** Representative transmission electron micrographs of Control (untreated) or *Ru1* (100μM)-treated Panc185 cells (24 h). Mitochondria are better defined in the Control compared to *Ru1*-treated samples. **F** Amount of *Ru1* molecules per 1000bp of DNA, determined by ICP, in mtDNA isolated from gradient-purified mitochondria from untreated (CTL) or *Ru1*-treated (100μM, 2 h) Panc185 cells.

Region	ID	POSITION	LENGTH	SCORE	ABS_SCORE	SEQUENCE	SUB_SCORE
D-loop	1	283	49	-1.2245	1.2245	ACAAAAAATTTCCACCAAACCCCCCCTCCCCCGCTTCTGGCCACAGCAC	-1.32,-1.48,-1.44,-1.6,-1.76,-1.92,-2.08,-2.24,-2.2,-2.24,-2.24,-2.24,-2.2,-2.12,-2.04,-1.88,-1.88,-1.96,-1.96,-2.0,-1.84,-1.64,-1.52,-1.36,-1.24
	2	431	30	-1.5333	1.5333	ACCCCCCAACTAACACATTATTTTCCCCTC	-1.24,-1.4,-1.4,-1.4,-1.24,-1.2
	3	442	40	-1.0000	1.0000	AACACATTATTTTCCCCTCCCACTCCCATACTACTAATCT	-1.24,-1.36,-1.48,-1.44,-1.44,-1.4,-1.44,-1.44,-1.44,-1.48,-1.48,-1.48,-1.48,-1.48,-1.36,-1.2
	4	477	44	-1.0909	1.0909	AATCTCATCAATACAACCCCCGCCCATCCTACCCAGCACACACA	-1.28,-1.28,-1.28,-1.36,-1.4,-1.4,-1.36,-1.48,-1.6,-1.68,-1.68,-1.64,-1.68,-1.68,-1.68,-1.68,-1.72,-1.56,-1.44,-1.28
	5	517	66	-1.2879	1.2879	CACACACCGCTGCTAACCCCATACCCCGAACCAACCAAACCCCAAAGACACCCCCCACAGTTTATG	-1.24,-1.36,-1.52,-1.44,-1.44,-1.4,-1.48,-1.48,-1.4,-1.44,-1.48,-1.56,-1.6,-1.56,-1.56,-1.72,-1.88,-1.88,-1.88,-1.72,-1.56,-1.56,-1.52,-1.52,-1.4,-1.24,-1.24,-1.24,-1.44,-1.6,-1.76,-1.84,-1.76,-1.8,-1.8,-1.68,-1.6,-1.6,-1.6,-1.6,-1.44,-1.24
RNR1	6	940	42	-0.8333	0.8333	GAGTGTTTTAGATCACCCCCTCCCCAATAAAGCTAAAACTCA	-1.32,-1.36,-1.36,-1.4,-1.4,-1.44,-1.44,-1.4,-1.44,-1.44,-1.44,-1.48,-1.48,-1.48,-1.48,-1.48,-1.36,-1.2
	7	1219	27	-1.1111	1.1111	AAACCCCGATCAACCTCACCACCTCTT	-1.2,-1.2,-1.2
RNR2	8	1670	38	-1.1053	1.1053	GCTAAACCTAGCCCCAAACCCACTCCACCTTACTACCA	-1.24,-1.36,-1.32,-1.4,-1.48,-1.48,-1.48,-1.4,-1.36,-1.36,-1.36,-1.48,-1.4,-1.24
	9	2053	29	-1.1379	1.1379	TTGCCCACAGAACCCTCTAAATCCCCTTG	-1.2,-1.36,-1.36,-1.4,-1.24
	10	3147	25	-1.2000	1.2000	CCTACTTCACAAAGCGCCTTCCCCC	-1.2
ND1	11	3473	27	-1.1852	1.1852	CACCAAAGAGCCCCTAAAACCCGCCAC	-1.24,-1.2,-1.24
	12	3481	28	-1.1786	1.1786	AGCCCCTAAAACCCGCCACATCTACCAT	-1.24,-1.32,-1.36,-1.2
	13	3504	33	-1.1818	1.1818	ACCATCACCCTCTACATCACCGCCCCGACCTTA	-1.28,-1.44,-1.32,-1.24,-1.32,-1.4,-1.36,-1.36,-1.24
	14	3548	57	-1.3684	1.3684	CGCTCTTCTACTATGAACCCCCCTCCCCATACCCAACCCCCTGGTCAACCTCAACCT	-1.24,-1.36,-1.56,-1.68,-1.68,-1.64,-1.64,-1.76,-1.84,-1.96,-1.96,-1.92,-2.08,-2.24,-2.4,-2.6,-2.76,-2.76,-2.52,-2.28,-2.12,-2.0,-1.84,-1.68,-1.76,-1.68,-1.52,-1.4,-1.24,-1.24,-1.32,-1.4,-1.28
	15	3880	29	-1.1379	1.1379	AGACCAACCGAACCCCCTTCGACCTTGCC	-1.2,-1.2,-1.2,-1.28,-1.28
	16	3959	25	-1.2000	1.2000	CCCCTTCGCCCTATTCTTCATAGCC	-1.2
	17	4227	26	-1.3462	1.3462	CCCATTACAATCTCCAGCATTCCCCC	-1.24,-1.28
	18	4235	28	-1.0714	1.0714	AATCTCCAGCATTCCCCCTCAAACCTAA	-1.2,-1.2,-1.2,-1.2
	19	4372	27	-1.1852	1.1852	TCCGTGCCACCTATCACACCCCATCCT	-1.2,-1.28,-1.2
	20	4453	27	-1.2593	1.2593	TACCCTTCCCGTACTAATTAATCCCCT	-1.2,-1.36,-1.36
	21	4460	25	-1.2000	1.2000	CCCGTACTAATTAATCCCCTGGCCC	-1.2
	22	4465	26	-1.1538	1.1538	ACTAATTAATCCCCTGGCCCAACCCG	-1.24,-1.2
	23	4468	32	-1.0000	1.0000	AATTAATCCCCTGGCCCAACCCGTCATCTACT	-1.2,-1.2,-1.2,-1.24,-1.24,-1.24,-1.28,-1.28
ND2	24	5189	33	-1.2424	1.2424	ACACCCTTAATTCCATCCACCCTCCTCTCCCTA	-1.24,-1.24,-1.24,-1.24,-1.24,-1.24,-1.24,-1.24,-1.24
	25	5293	25	-1.2000	1.2000	CCTCATCATCCCCACCATCATAGCC	-1.2
	26	5296	25	-1.2000	1.2000	CATCATCCCCACCATCATAGCCACC	-1.2
	27	5299	25	-1.2000	1.2000	CATCCCCACCATCATAGCCACCATC	-1.2
	28	5301	28	-1.3571	1.3571	TCCCCACCATCATAGCCACCATCACCCT	-1.28,-1.4,-1.36,-1.2
	29	5362	25	-1.2000	1.2000	CTCCACCTCAATCACACTACTCCCC	-1.2
	30	5420	51	-1.2157	1.2157	GAACATACAAAACCCACCCCATTCCTCCCCACACTCATCGCCCTTACCACG	-1.2,-1.24,-1.4,-1.56,-1.68,-1.84,-1.84,-1.88,-1.84,-1.88,-1.88,-1.92,-1.92,-1.8,-1.72,-1.56,-1.68,-1.64,-1.6,-1.44,-1.28,-1.28,-1.36,-1.44,-1.36,-1.32,-1.28
	31	5466	25	-1.2400	1.2400	CCACGCTACTCCTACCTATCTCCCC	-1.24
	32	5874	28	-1.1429	1.1429	CACTCAGCCATTTTACCTCACCCCCACT	-1.24,-1.2,-1.24,-1.2
COX1	33	6148	29	-1.0345	1.0345	AGTTCCCCTAATAATCGGTGCCCCCGATA	-1.24,-1.2,-1.24,-1.24,-1.24
	34	6166	27	-1.1111	1.1111	TGCCCCCGATATGGCGTTTCCCCGCAT	-1.2,-1.2,-1.24
	35	6409	36	-1.0556	1.0556	CAATATAAAACCCCCTGCCATAACCCAATACCAAAC	-1.2,-1.28,-1.28,-1.28,-1.28,-1.28,-1.36,-1.44,-1.44,-1.44,-1.44,-1.32
	36	6425	32	-1.0313	1.0313	GCCATAACCCAATACCAAACGCCCCTCTTCGT	-1.28,-1.32,-1.28,-1.2,-1.2,-1.24,-1.2,-1.2
	37	6544	25	-1.2400	1.2400	CCTCAACACCACCTTCTTCGACCCC	-1.24
	38	6547	25	-1.2000	1.2000	CAACACCACCTTCTTCGACCCCGCC	-1.2
	39	7384	39	-0.8718	0.8718	ACTATATGGATGCCCCCCACCCTACCACACATTCGAAGA	-1.24,-1.32,-1.28,-1.32,-1.32,-1.36,-1.36,-1.36,-1.44,-1.56,-1.52,-1.52,-1.56,-1.36,-1.2
COX2	40	7800	32	-1.1250	1.1250	CCTAGTCCTCATCGCCCTCCCATCCCTACGCA	-1.28,-1.32,-1.24,-1.24,-1.28,-1.28,-1.32,-1.24
	41	7809	31	-1.0968	1.0968	CATCGCCCTCCCATCCCTACGCATCCTTTAC	-1.24,-1.28,-1.28,-1.28,-1.24,-1.28,-1.2
	42	7942	26	-1.1538	1.1538	TCCTACATACTTCCCCCATTATTCCT	-1.2,-1.2
	43	8250	28	-1.2500	1.2500	GCCCGTATTTACCCTATAGCACCCCCTC	-1.2,-1.48,-1.36,-1.28
	44	8255	45	-1.2889	1.2889	TATTTACCCTATAGCACCCCCTCTACCCCCTCTAGAGCCCACTGT	-1.2,-1.36,-1.52,-1.68,-1.84,-2.0,-2.0,-1.92,-1.8,-1.68,-1.64,-1.64,-1.6,-1.72,-1.88,-1.96,-1.96,-1.84,-1.68,-1.48,-1.32
ATP8	45	8385	25	-1.2000	1.2000	CGTATGGCCCACCATAATTACCCCC	-1.2
	46	8389	30	-1.1333	1.1333	TGGCCCACCATAATTACCCCCATACTCCTT	-1.2,-1.2,-1.36,-1.52,-1.4,-1.28
	47	8396	25	-1.2000	1.2000	CCATAATTACCCCCATACTCCTTAC	-1.2
	48	8404	26	-1.1923	1.1923	ACCCCCATACTCCTTACACTATTCCT	-1.24,-1.24
	49	8462	29	-1.1724	1.1724	ACCACCTACCTCCCTCACCAAAGCCCATA	-1.24,-1.36,-1.28,-1.2,-1.2
ATP6	50	8554	29	-1.2414	1.2414	TTGCCCCCACAATCCTAGGCCTACCCGCC	-1.2,-1.32,-1.28,-1.4,-1.32
	51	8596	36	-1.2500	1.2500	TTCTATTTCCCCCTCTATTGATCCCCACCTCCAAAT	-1.32,-1.48,-1.48,-1.52,-1.6,-1.6,-1.68,-1.76,-1.76,-1.6,-1.44,-1.28
	52	8917	39	-0.8974	0.8974	AAGGCACACCTACACCCCTTATCCCCATACTAGTTATTA	-1.24,-1.4,-1.4,-1.48,-1.56,-1.56,-1.56,-1.52,-1.48,-1.4,-1.32,-1.32,-1.32,-1.28,-1.28
COX3	53	9233	30	-1.0667	1.0667	ATAGTAAAACCCAGCCCATGACCCCTAACA	-1.24,-1.24,-1.24,-1.24,-1.32,-1.32
	54	9504	25	-1.2400	1.2400	CCTTTTACCACTCCAGCCTAGCCCC	-1.24
	55	9507	39	-1.1282	1.1282	TTTACCACTCCAGCCTAGCCCCTACCCCCCAATTAGGAG	-1.24,-1.4,-1.56,-1.72,-1.88,-1.96,-1.88,-1.88,-1.84,-1.84,-1.76,-1.6,-1.52,-1.56,-1.36
	56	9549	31	-1.0323	1.0323	ACTGGCCCCCAACAGGCATCACCCCGCTAAA	-1.28,-1.24,-1.24,-1.24,-1.32,-1.4,-1.24
	57	9560	27	-1.1481	1.1481	ACAGGCATCACCCCGCTAAATCCCCTA	-1.24,-1.24,-1.2
	58	9564	33	-1.2121	1.2121	GCATCACCCCGCTAAATCCCCTAGAAGTCCCAC	-1.24,-1.32,-1.24,-1.24,-1.36,-1.44,-1.56,-1.4,-1.28
ND3	59	10174	44	-1.0909	1.0909	CGGCTTCGACCCTATATCCCCCGCCCGCGTCCCTTTCTCCATAA	-1.28,-1.36,-1.4,-1.52,-1.44,-1.44,-1.56,-1.64,-1.8,-1.8,-1.68,-1.56,-1.48,-1.48,-1.56,-1.64,-1.64,-1.64,-1.48,-1.32
	60	10260	36	-1.1111	1.1111	AAATTGCCCTCCTTTTACCCCTACCATGAGCCCTAC	-1.28,-1.28,-1.28,-1.24,-1.24,-1.2,-1.36,-1.36,-1.36,-1.24,-1.24,-1.2
ND4L	61	10604	33	-1.0909	1.0909	CTCATAACCCTCAACACCCACTCCCTCTTAGCC	-1.28,-1.24,-1.28,-1.24,-1.24,-1.24,-1.2,-1.28,-1.24
ND4	62	10911	60	-1.3500	1.3500	CTGTTCCCCAACCTTTTCCTCCGACCCCCTAACAACCCCCCTCCTAATACTAACTACCTG	-1.24,-1.36,-1.52,-1.72,-1.88,-1.88,-1.72,-1.56,-1.44,-1.28,-1.28,-1.44,-1.52,-1.6,-1.76,-1.92,-2.08,-2.08,-2.08,-2.08,-2.08,-2.0,-1.92,-1.96,-1.96,-1.84,-1.68,-1.52,-1.36,-1.24,-1.24,-1.24,-1.32,-1.36,-1.36,-1.32
	63	11212	29	-1.1034	1.1034	TACACCCTAGTAGGCTCCCTTCCCCTACT	-1.24,-1.24,-1.24,-1.24,-1.24
	64	11226	25	-1.2000	1.2000	CTCCCTTCCCCTACTCATCGCACTA	-1.2
	65	11405	27	-1.2963	1.2963	TCCCTAAAGCCCATGTCGAAGCCCCCA	-1.24,-1.4,-1.28
	66	11491	25	-1.2000	1.2000	CGCCTCACACTCATTCTCAACCCCC	-1.2
	67	11530	27	-1.1852	1.1852	GCCTACCCCTTCCTTGTACTATCCCTA	-1.28,-1.32,-1.24
	68	11652	26	-1.1538	1.1538	AACAGCCATTCTCATCCAAACCCCCT	-1.2,-1.2
	69	11846	40	-1.0500	1.0500	GCCTCGCTAACCTCGCCTTACCCCCCACTATTAACCTACT	-1.28,-1.48,-1.4,-1.36,-1.36,-1.32,-1.36,-1.32,-1.32,-1.32,-1.4,-1.4,-1.32,-1.32,-1.32,-1.36
	70	12062	26	-1.3462	1.3462	CCCTCATGTTCATACACCTATCCCCC	-1.24,-1.28
	71	12070	51	-1.2157	1.2157	TTCATACACCTATCCCCCATTCTCCTCCTATCCCTCAACCCCGACATCATT	-1.24,-1.24,-1.32,-1.36,-1.36,-1.36,-1.36,-1.44,-1.56,-1.6,-1.52,-1.56,-1.56,-1.56,-1.56,-1.56,-1.56,-1.56,-1.36,-1.36,-1.4,-1.4,-1.36,-1.4,-1.32,-1.24,-1.24
	72	12099	25	-1.2400	1.2400	ATCCCTCAACCCCGACATCATTACC	-1.24
ND5	73	12354	29	-1.0690	1.0690	ATAACCACCCTAACCCTGACTTCCCTAAT	-1.24,-1.24,-1.24,-1.24,-1.24
	74	12360	54	-1.4259	1.4259	ACCCTAACCCTGACTTCCCTAATTCCCCCCATCCTTACCACCCTCGTTAACCCT	-1.24,-1.4,-1.44,-1.48,-1.52,-1.68,-1.68,-1.68,-1.64,-1.6,-1.48,-1.48,-1.52,-1.6,-1.64,-1.64,-1.76,-1.76,-1.76,-1.64,-1.68,-1.64,-1.64,-1.64,-1.64,-1.48,-1.44,-1.4,-1.36,-1.2
	75	12543	26	-1.1538	1.1538	ACAACCCAAACAACCCAGCTCTCCCT	-1.2,-1.2
	76	13013	37	-0.9730	0.9730	ATTAGGTCTCCACCCCTGACTCCCCTCAGCCATAGAA	-1.32,-1.32,-1.36,-1.36,-1.32,-1.48,-1.64,-1.64,-1.6,-1.6,-1.48,-1.4,-1.4
	77	13031	37	-1.3243	1.3243	ACTCCCCTCAGCCATAGAAGGCCCCACCCCAGTCTCA	-1.28,-1.28,-1.4,-1.56,-1.56,-1.56,-1.4,-1.2,-1.2,-1.2,-1.2,-1.28,-1.2
	78	13045	37	-1.1622	1.1622	TAGAAGGCCCCACCCCAGTCTCAGCCCTACTCCACTC	-1.2,-1.32,-1.44,-1.48,-1.48,-1.52,-1.6,-1.76,-1.68,-1.52,-1.4,-1.24,-1.28
	79	13107	27	-1.1481	1.1481	TTCTTACTCATCCGCTTCCACCCCCTA	-1.24,-1.24,-1.24
	80	13111	26	-1.1538	1.1538	TACTCATCCGCTTCCACCCCCTAGCA	-1.2,-1.2
	81	13123	27	-1.1852	1.1852	TCCACCCCCTAGCAGAAAATAGCCCAC	-1.24,-1.24,-1.2
	82	13633	31	-1.0000	1.0000	GTCAACCTCGCTTCCCCACCCTTACTAACAT	-1.2,-1.28,-1.28,-1.24,-1.28,-1.28,-1.2
	83	13667	39	-1.1795	1.1795	CGAAAATAACCCCACCCTACTAAACCCCATTAAACGCCT	-1.2,-1.32,-1.52,-1.68,-1.68,-1.68,-1.68,-1.68,-1.68,-1.68,-1.56,-1.36,-1.28,-1.2,-1.2
	84	13739	55	-1.3455	1.3455	TACTAACAACATTTCCCCCGCATCCCCCTTCCAAACAACAATCCCCCTCTACCTA	-1.24,-1.4,-1.56,-1.68,-1.68,-1.68,-1.76,-1.8,-1.8,-1.8,-1.76,-1.8,-1.8,-1.8,-1.84,-1.68,-1.52,-1.36,-1.36,-1.36,-1.56,-1.68,-1.84,-1.84,-1.72,-1.56,-1.4,-1.32,-1.24,-1.24,-1.24
	85	13780	27	-1.3333	1.3333	TCCCCCTCTACCTAAAACTCACAGCCC	-1.2,-1.32,-1.28
	86	13968	27	-1.1481	1.1481	AGCCAAAACCTGCCCCTACTCCTCCTA	-1.24,-1.24,-1.28
	87	13974	27	-1.1481	1.1481	AACCTGCCCCTACTCCTCCTAGACCTA	-1.24,-1.24,-1.24
	88	13979	26	-1.1923	1.1923	GCCCCTACTCCTCCTAGACCTAACCT	-1.24,-1.28
ND6	89	14134	25	-1.2000	1.2000	TCCTAATCACATAACCTATTCCCCC	-1.2
	90	14223	25	-1.2000	1.2000	ACGCCCATAATCATACAAAGCCCCC	-1.2
	91	14226	25	-1.2000	1.2000	CCCATAATCATACAAAGCCCCCGCA	-1.2
	92	14242	26	-1.1923	1.1923	GCCCCCGCACCAATAGGATCCTCCCG	-1.28,-1.28
	93	14258	41	-1.0000	1.0000	GATCCTCCCGAATCAACCCTGACCCCTCTCCTTCATAAATT	-1.24,-1.48,-1.48,-1.52,-1.44,-1.44,-1.52,-1.4,-1.28,-1.2,-1.24,-1.24,-1.24,-1.24,-1.2,-1.2,-1.2
	94	14323	30	-1.0667	1.0667	TTTACCACAACCACCACCCCATCATACTCT	-1.2,-1.2,-1.24,-1.24,-1.28,-1.2
	95	14333	30	-1.2667	1.2667	CCACCACCCCATCATACTCTTTCACCCACA	-1.24,-1.28,-1.32,-1.32,-1.28,-1.2
	96	14367	25	-1.2400	1.2400	CAATCCTACCTCCATCGCTAACCCC	-1.24
	97	14369	27	-1.1111	1.1111	ATCCTACCTCCATCGCTAACCCCACTA	-1.2,-1.2,-1.2
	98	14401	42	-1.0952	1.0952	CTCACCAAGACCTCAACCCCTGACCCCCATGCCTCAGGATAC	-1.32,-1.44,-1.6,-1.72,-1.72,-1.64,-1.52,-1.6,-1.68,-1.72,-1.76,-1.68,-1.52,-1.44,-1.4,-1.4,-1.4,-1.28
	99	14509	35	-0.9714	0.9714	ACTATTAAACCCATATAACCTCCCCCAAAATTCAG	-1.2,-1.36,-1.32,-1.32,-1.32,-1.32,-1.32,-1.32,-1.36,-1.36,-1.2
	100	14607	40	-1.0750	1.0750	CTTAGAAGAAAACCCCACAAACCCCATTACTAAACCCACA	-1.28,-1.24,-1.24,-1.24,-1.24,-1.32,-1.32,-1.32,-1.36,-1.36,-1.48,-1.6,-1.72,-1.56,-1.44,-1.28
	101	14628	25	-1.2000	1.2000	CCCCATTACTAAACCCACACTCAAC	-1.2
CYTB	102	14748	29	-1.2759	1.2759	GACCCCAATACGCAAAACTAACCCCCTAA	-1.32,-1.52,-1.52,-1.36,-1.2
	103	14789	46	-1.1304	1.1304	ACCACTCATTCATCGACCTCCCCACCCCATCCAACATCTCCGCATG	-1.24,-1.4,-1.48,-1.56,-1.56,-1.52,-1.6,-1.64,-1.64,-1.64,-1.68,-1.64,-1.64,-1.68,-1.64,-1.76,-1.84,-1.72,-1.68,-1.68,-1.52,-1.32
	104	15361	30	-1.1667	1.1667	AACAACCCCCTAGGAATCACCTCCCATTCC	-1.24,-1.24,-1.24,-1.2,-1.28,-1.36
	105	15512	51	-1.3529	1.3529	ATACCCTAGCCAACCCCTTAAACACCCCTCCCCACATCAAGCCCGAATGAT	-1.32,-1.48,-1.64,-1.8,-1.68,-1.72,-1.76,-1.92,-2.08,-2.12,-2.08,-2.0,-2.0,-2.04,-1.88,-1.72,-1.52,-1.48,-1.6,-1.72,-1.68,-1.68,-1.68,-1.64,-1.6,-1.44,-1.28
D-loop	106	16162	47	-1.0851	1.0851	AAAACCCAATCCACATCAAAACCCCCTCCCCATGCTTACAAGCAAGT	-1.24,-1.4,-1.4,-1.56,-1.72,-1.76,-1.8,-1.68,-1.68,-1.64,-1.68,-1.6,-1.52,-1.52,-1.52,-1.52,-1.52,-1.44,-1.48,-1.48,-1.48,-1.44,-1.28
	107	16242	32	-0.9688	0.9688	TGCAACTCCAAAGCCACCCCTCACCCACTAGG	-1.24,-1.36,-1.4,-1.4,-1.4,-1.4,-1.28,-1.2
	108	16254	26	-1.1538	1.1538	GCCACCCCTCACCCACTAGGATACCA	-1.2,-1.24
	109	16352	35	-1.2857	1.2857	CCCTTCTCGTCCCCATGGATGACCCCCCTCAGATA	-1.32,-1.36,-1.4,-1.44,-1.44,-1.48,-1.44,-1.4,-1.36,-1.4,-1.4

Fig. S5 *GQ in the mtDNA determined with G4 hunter.*

A

Position 16,162, SCORE: -1.085
AAAACCCAATCCACATCAAAACCCCCTCCCCATGCTTACAAGCAAGT
Position 16,242, SCORE: -0.969
TGCAACTCCAAAGCCACCCCTCACCCACTAGG
Position 16,254; SCORE: -1.154
GCCACCCCTCACCCACTAGGATACCA
Position 16,352; SCORE: -1.286
CCCTTCTCGTCCCCATGGATGACCCCCCTCAGATA
Position 283, SCORE: -1.224
ACAAAAAATTTCCACCAAACCCCCCCTCCCCCGCTTCTGGCCACAGCAC
Position 431, SCORE: -1.533
ACCCCCCAACTAACACATTATTTTCCCCTC
Position 442; SCORE: -1.0
AACACATTATTTTCCCCTCCCACTCCCATACTACTAATCT
Position 477, SCORE: -1.091
AATCTCATCAATACAACCCCCGCCCATCCTACCCAGCACACACA
Position 517, SCORE: -1.288
CACACACCGCTGCTAACCCCATACCCCGAACCAACCAAACCCCAAAGA
CACCCCCACAGTTTATG

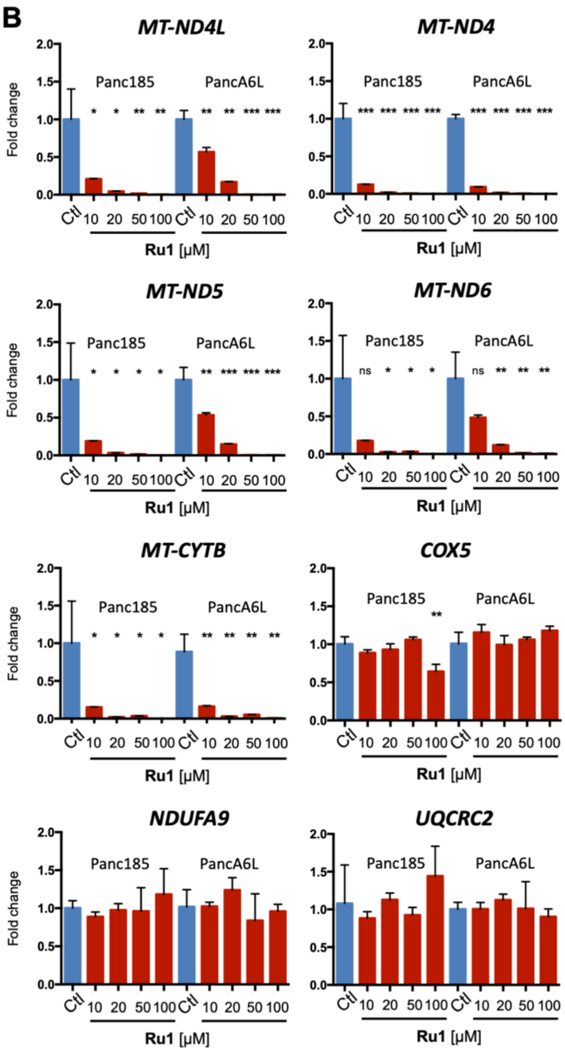

Fig. S6 *Ru1 inhibits mtDNA transcription.* **A** Sequence of 9 predicted GQs in the D-loop region, their positions and G4Hunter score indicating G4-prone structures. Isolated guanines (**G**) are shown in red, and cysteines (**C**) in blue. **B** Mean fold-change ± SD in the relative mRNA expression of the indicated mtDNA- and nuclear-encoded genes as a function of increasing concentrations of *Ru1* in Panc185 or PanA6L cells (48h treatment). Values were normalized to ß-actin levels. * $p < 0.05$, ** $p < 0.01$, *** $p < 0.001$, as determined by one-way ANOVA with Dunnett post-test, compared to untreated (Ctl).

SUPPLEMENTARY TABLES

Table S1 *Antibodies*

1ª Abs-Epitope	Source	Dilution	Application	Manufacturer (Catalog no.)
α-hu/mu-GAPDH	Mouse monoclonal	1:5000	WB	Abcam (Cat no. ab8245)
Total OXPHOS Human WB Antibody Cocktail	Mouse monoclonal	1:500	WB	Abcam (Cat no. ab110411)
α-hu-CD133/1-APC	Mouse monoclonal	1:50	FC	Miltenyi Biotec (Cat no. 130-090-826)
α-hu-EpCAM-FITC	Mouse monoclonal	1:50	FC	Miltenyi (Cat no. 130-110-998)
α-hu-CXCR4-PE	Mouse monoclonal	1:50	FC	Miltenyi (Cat no. 130-117-354)
α-hu-CD90-APC	Mouse monoclonal	1:20	FC	Molecular Probes (Cat no. A15726)

2ª Abs-Epitope	Source	Dilution	Application	Manufacturer (Catalog no.)
Anti-mouse-HRP	Sheep	1:5000	WB	Amersham (NA931)
Anti-rabbit-HRP	Donkey	1:5000	WB	Amersham (NA934)

Table S2 *RTqPCR primer sequences*

Gene	Species	Primer sense	Primer antisense
β-ACTIN	human	GCGAGCACACGAGCCTCGCCTT	CATCATCCATGGTGAGCTGGCGG
POU5F1	human	CTTGCTGCAGAAGTGGGTGGAGGAA	CTGCAGTGTGGGTTTCGGGCA
SOX2	human	AGAACCCCAAGATGCACAAC	CGGGGCCGGTATTTATAATC
PGC1A	human	TGACTGGCGTCATTCAGGAG	CCAGAGCAGCACACTCGAT
MT-ND1	human	GCACTGCGAGCAGTAGCCCA	TGGCCAAGGGTCATGATGGCA
MT-ND2	human	AACTCCAGCACCACGACCCT	AAAAGCCGGTTAGCGGGGGC
MT-CO1	human	CTCTTCGTCTGATCCGTCCT	ATTCCGAAGCCTGGTAGGAT
MT-CO2	human	CGCCCTCCCATCCCTACGCA	CCGCCGTAGTCGGTGTACTCG
MT-ATP8	human	CCACCTACCTCCCTCACCAAAGC	TGGGGGCAATGAATGAAGCGAAC
MT-ATP6	human	TCCCCTTATGAGCGGGCACAG	TAGGCGTACGGCCAGGGCTA
MT-CO3	human	GCCCTCCTAATGACCTCCGGC	TGGACAGGTGGTGTGTGGTGG
MT-ND3	human	TCGACCCTATATCCCCCGCCC	TGGTAGGGGTAAAAGGAGGGCA
MT-ND4L	human	TCGCTCACACCTCATATCCTCCCTA	AGAGGGAGTGGGTGTTGAGGGTT
MT-ND4	human	TCGCCCACGGGCTTACATCC	AGGCGAGGTTAGCGAGGCTT
MT-ND5	human	TCCGCTTCCACCCCCTAGCA	GGCGCAGACTGCTGCGAACA
MT-ND6	human	GATTGTTAGCGGTGTGGTCGGGT	GACCTCAACCCCTGACCCCCA
MT-CYTB	human	ACCAGACGCCTCAACCGCC	GCCTCGCCCGATGTGTAGGAA
COX5	human	CTTTGCGGGCATGCAGACGG	AGCCCATCCATGCGGTTTACACT
NDUFA9	human	AGTGGAGCGGATGCACATCACA	GACGGTCTTGGCCGGCTTCA
UQCRC2	human	GGCCACAGCTGCTGGAGATGTTA	GCAACTAGAGCCTGGGACCCG
Hprt	mouse	TCCTCCTCAGACCGCTTTT	CCTGGTTCATCATCGCTAATC
Atp6	mouse	TCCCAATCGTTGTAGCCATCA	AGACGGTTGTTGATTAGGCGT
Cox1	mouse	ATCACTACCAGTGCTAGCCG	CCTCCAGCGGGATCAAAGAA
Drp1	mouse	ATGCCAGCAAGTCCACAGAA	TGTTCTCGGGCAGACAGTTT

Table S3 *PCR primer sequences*

Gene	Species	Primer sense	Primer antisense
D-loop	human	CTCACCCATCAACAACCGCT	TATGGGGTGATGTGAGCCCG
MT-RNR2	human	TTCAAGCTCAACACCCACTACC	GGAGCCATTCATACAGGTCCCTATT
